# Controlling the Kinetic and Electrochemical Properties of Eu^II^–Containing Complexes Using Peripheral Charges

**DOI:** 10.1021/acs.inorgchem.5c05456

**Published:** 2026-01-24

**Authors:** Md Sydul Islam, Matthew J. Allen

**Affiliations:** Department of Chemistry, 2954 Wayne State University, Detroit, Michigan 48201, United States

## Abstract

The development of Eu-based contrast agents for magnetic resonance imaging requires careful balance between electrochemical potential and inertness with respect to metal dissociation. Using coordination chemistry, this study aimed to understand how the distance between peripheral anionic groups and Eu^II^ in macrocyclic complexes affects these properties. Four Eu^II^-containing complexes were synthesized with different maximum-possible distances between the Eu^II^ ion and peripheral anionic groups of the ligand. Electrochemical potentials were measured using cyclic voltammetry, and dissociation rates were measured at pH 7 using an electrochemical method and at pH 1 using an acid-catalyzed dissociation method. Electrochemical studies show that increasing the maximum-possible distance between Eu^II^ and peripheral charges shifts electrochemical potentials to more positive values. Additionally, a decrease in the dissociation rates was observed at pH 7 with increasing maximum-possible distance. The Eu^II^-containing complex with the largest maximum-possible distance exhibited the slowest dissociation rate. Statistical analysis confirmed that trends are significant. The findings are expected to provide a framework for the rational design of Eu^II^-based contrast agents for magnetic resonance imaging in which redox accessibility and kinetic inertness can be fine-tuned independently by controlling the distance of peripheral charges from Eu^II^.

## Graphical abstract

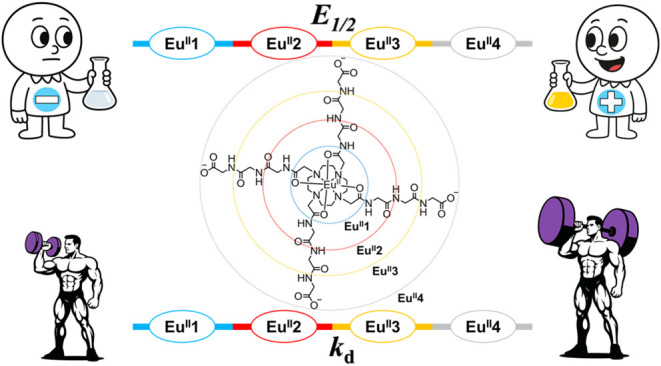

## 1 Introduction

Magnetic resonance imaging (MRI) is a noninvasive imaging method that is ubiquitous in clinical diagnostics. MRI is often complemented by paramagnetic contrast agents that relax the nuclear spins of nearby protons, enhancing the contrast of images. Advancements in contrast agents for MRI include modifications of contrast agents to provide functional information in addition to anatomical information, for example, reporting physiological and pathological conditions, including hypoxia, pH, redox dynamics, and metabolic fluxes.^1–4^ Among these developments, contrast agents, including those containing divalent europium (Eu^II^), have shown great promise for imaging hypoxia,^5^ which is linked with diseases including cancer,^6^ ischemic heart disease,^7^ and stroke.^8^ Eu^II^ is isoelectronic with Gd^III^, and both ions serve as positive contrast agents in *T*_1_-weighted MRI.^9^ Moreover, Eu^II^ undergoes one-electron oxidation to form Eu^III^ that does not enhance contrast,^10^ and the complete loss of positive contrast enhancement following oxidation of Eu^II^ provides a distinctive platform for oxygen sensing, where Eu^II^ enhances contrast in oxygen-depleted, or hypoxic, environments but not in normoxic environments.^11,12^ Most in vivo studies involving Eu^II^ require direct injection into a site of interest;^4,12,13^ however, recent developments focused on increasing the persistence of Eu^II^ in vivo using kinetic strategies led to relatively long-lasting contrast agents.^14,15^ With a long enough persistence in oxygenated solutions, including blood, Eu^II^-containing contrast agents would become compatible with the multitude of targeting strategies developed for other imaging probes.^16^ One of the strategies to increase the persistence of Eu^II^-containing contrast agents in vivo involves the inclusion of phosphonates on the periphery of the ligand.^15^ These charged groups enable the enhanced persistence times, but they also raise questions regarding the effect of peripheral charges on other aspects of coordination chemistry relevant to contrast agents. Specifically, peripheral charges likely influence the redox potential of the Eu^II/III^ couple and the inertness of the complex from demetalation.

For in vivo hypoxia imaging, it is crucial that europium-based contrast agents (1) have an electrochemical potential between that of water and O_2_ reduction and (2) that they are inert from dissociation from metal ions. The Eu^II/III^ redox couple should ideally fall between that of water and oxygen, and maintaining inertness from demetalation is critical because dissociation would lead to immediate loss of signal enhancement from oxidation of Eu^II^ to Eu^III^.^10^ To systematically study the impact of peripheral charge on redox potential and inertness, we sought to test the hypotheses that (1) anionic charges not directly coordinated to Eu^II^ will have minimal influence on electrochemical potential because of electrostatic stabilization of the divalent state and (2) the addition of anionic charges on the periphery of Eu^II^-containing complexes influences dissociation as a function of distance from Eu^II^ because of electrostatic interactions. To test these hypotheses, we synthesized and characterized a series of four Eu^II^-containing macrocyclic complexes with different maximum-possible distances between Eu^II^ ions and peripheral anionic groups (Figure 1), and we examined the electrochemical behavior and dissociation rates of these complexes. The results of these studies are reported here along with a discussion of how the findings are relevant to the design of Eu^II^-containing contrast agents.

**Figure 1:**
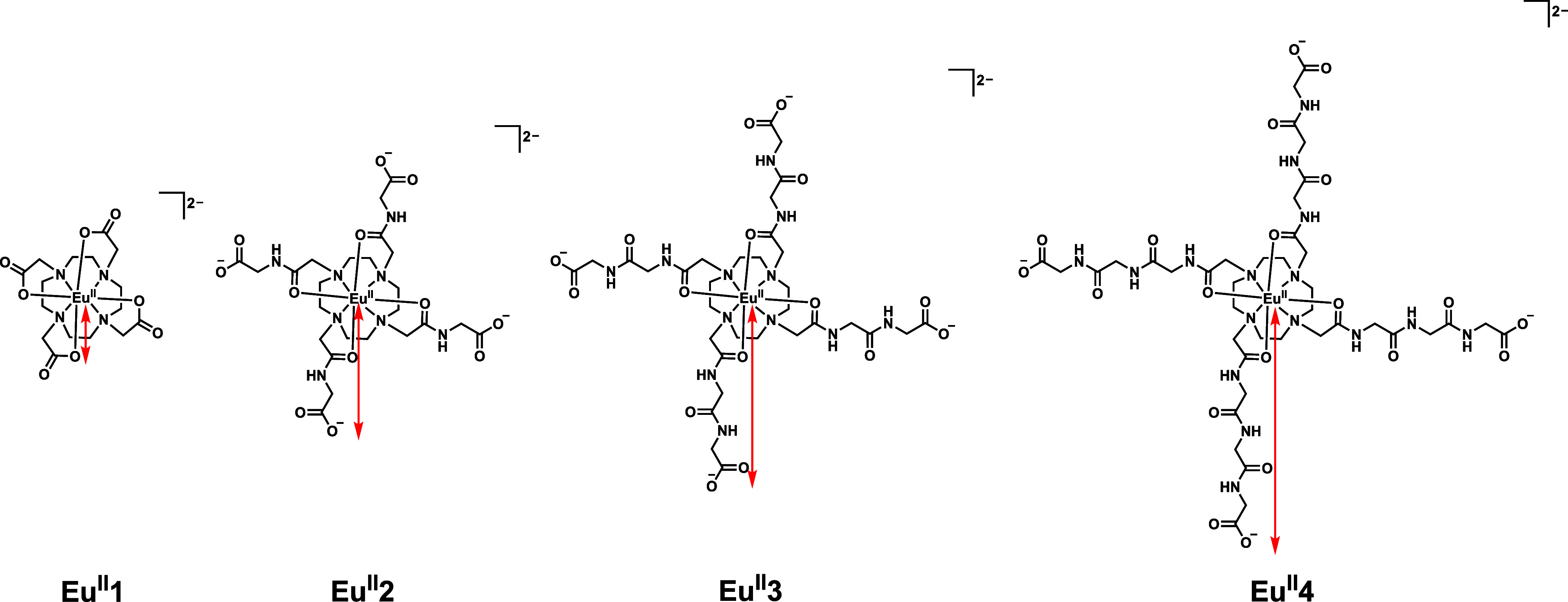
Complexes reported in this manuscript. Na^+^ counterions are not shown for clarity. Red arrows denote the maximum-possible distances between Eu^II^ and peripheral ligand charges.

## 2 Experimental Section

### 2.1 Materials

Commercially available chemicals were of reagent-grade purity or better and were used without purification unless otherwise noted. 1,4,7,10-Tetraazacyclododecane (cyclen) was purchased from Strem Chemicals. 1,4,7,10-Tetraazacyclododecane-1,4,7,10-tetraacetic acid (**1**) and europium­(III) 1,4,7,10-tetraazacyclododecane-1,4,7,10-tetrakis­(acetamidoacetic acid) (**Eu** ^**III**^ **2**) were purchased from Macrocyclics. The Eu^III^-containing complex of **1**,^17^ the Eu^II^-containing complex of **2**,^10^ and ethyl (2-chloroacetyl)­glycylglycinate (**5**)^18^ were synthesized following reported procedures. EuCl_2_, EuCl_3_·6H_2_O, ethyl glycylglycinate hydrochloride, ethyl glycylglycylglycinate hydrochloride, chloroacetyl chloride, Dowex 50WX8 (hydrogen form) resin, zinc dust, and K_2_CO_3_ were purchased from Sigma-Aldrich. Tetraethylammonium perchlorate was purchased from Alfa Aesar. All deuterated solvents were purchased from Cambridge Isotope Laboratories. Water was purified using a PURELAB Ultra Mk2 water purification system (ELGA). DOWEX-Na^+^ was prepared as previously reported.^10^ Activated Zn^0^ was prepared using a reported method.^19^ Samples containing divalent europium were prepared in a wet glovebox (water allowed but no O_2_) under an atmosphere of N_2_.

### 2.2 Instrumentation

^1^H NMR and ^13^C NMR spectra were obtained using a Bruker Avance NEO (500 MHz for ^1^H and 126 MHz for ^13^C) spectrometer. Chemical shifts are reported relative to residual solvent signals [(CD_3_)_2_SO: ^1^H, 2.50, ^13^C, 39.53; D_2_O: ^1^H, 4.79]. NMR data are assumed to be first-order, and the apparent multiplicity is reported as “s” = singlet, “d” = doublet, “t” = triplet, “q” = quartet, and “td” = triplet of doublets. Italicized elements are those that are responsible for the shifts. Correlation spectroscopy, distortionless enhancement by polarization transfer, and heteronuclear single quantum coherence spectra were used to assign spectral peaks.

Elemental analyses (C, H, N, and Cl; reported as percentages) were performed by Midwest Microlab (Indianapolis, IN). Thermogravimetric analyses were performed using a TGA Q-50 apparatus (TA Instruments) situated in an Ar-filled glovebox with a ramp of 10 °C/min. High-resolution mass spectrometry was performed using a Thermo LTQ-Orbitrap XL ESI mass spectrometer in the Lumigen Instrument Center at Wayne State University.

A Fisher Scientific Centrific Centrifuge (Model 225, 1000 rpm) was used to separate excess europium as Eu­(OH)_3_ during the work up of metalation reaction. Filtration involved Whatman filter paper (Grade 2) or Millex hydrophilic polytetrafluoroethylene syringe filters (0.2 μm pore size). MQuant pH-indicator strips were used to monitor pH.

UV–visible absorbance spectra were collected using a Carey 60 spectrophotometer, and samples were loaded in quartz cuvettes under an atmosphere of N_2_. Luminescence spectra were recorded using a HORIBA Jobin Yvon Fluoromax-4 spectrofluorometer. An emission range of 550–725 nm was recorded using an excitation wavelength of 395 nm (1 nm slit widths and 1 nm resolution).

Concentrations of Eu were determined using energy-dispersive X-ray fluorescence (ED-XRF) spectroscopy at the Lumigen Instrument Center in the Department of Chemistry at Wayne State University. Calibration curves were created using the ^153^Eu isotope ion count for a concentration range of 125–1000 ppm (diluted from Sigma ICP standard solution).

### 2.3 Minimum Detectable Concentration

The minimum detectable concentrations of **Eu** ^**III**^ **1**, **Eu** ^**III**^ **3**, and **Eu** ^**III**^ **4** were determined by adopting a reported procedure.^20^ Briefly, the absolute emission intensity at 587 nm (excitation at 395 nm) was measured as a function of the concentration of Eu^III^L (L = **1**, **3** or **4**) (Figure S6). The emission intensity of the least concentrated sample of Eu^III^L (0.03125 mM) was measured using seven independently prepared samples. The minimum detectable concentration values were determined using eq 1, in which σ is the standard deviation and *m* is the slope of the best fit line in a plot of emission intensity versus concentration.

1 minimumdetectableconcentration=3σ/m

### 2.4 Cyclic Voltammetry

Cyclic voltammetry involved a three-electrode system comprised of a glassy-carbon working electrode, a Pt-wire auxiliary electrode, and a Ag/AgCl reference electrode coupled with a Pine Wavenow USB or a BAS 50W potentiostat. Tetraethylammonium perchlorate was used as the electrolyte. The glassy-carbon electrode was polished using a slurry of alumina powder (0.05 μm) with deionized water on a polishing cloth three times before every experiment. Acquisition parameters for the cyclic voltammograms of all complexes were eight sweeps, an upper potential of 0 V, a lower potential of −1.5 V, an initial potential of −1.5 V (rising), a final potential of −1.5 V, and a sweep rate of 100 mV s^–1^. Samples were prepared by dissolving Eu^III^-containing complexes (3 mM) with tetraethylammonium perchlorate (50 mM) in water. The pH of the resulting solution was adjusted to 7 using aqueous NaOH (1 M). Solutions were sparged with Ar for 5–10 min while stirring before measurements were performed without sparging or stirring. From the voltammograms, electrochemical potentials were calculated using eq 2, in which *E*_pc_ and *E*_pa_ are the cathodic and anodic peak potentials, respectively. Electrochemical potentials are reported as mean ± standard error of the mean of three independently prepared samples.

2 $$E_{1/2}=\frac{E_{\mathrm{pc}}+E_{\mathrm{pa}}}{2}$$

### 2.5 Dissociation Kinetic Measurements

The dissociation kinetics data for **Eu** ^**II**^ **1**–**Eu** ^**II**^ **4** at pH 1 were acquired by adopting an acid-catalyzed dissociation method.^10^ Briefly, aqueous degassed HCl (0.1 M) was used to bring the pH of separate solutions of each complex (6 mM in degassed water) to 1, and dissociation was monitored using UV–visible spectroscopy at 420 nm. Samples were loaded into quartz cuvettes sealed using airtight caps and paraffin wax under an atmosphere of N_2_ before spectrophotometry measurements. Plots of absorbance as a function of time were used to calculate dissociation rate constants (*k*_d_) using eq 3, in which *k*_d_ is the calculated first-order dissociation rate constant; *t* is time; and *A*_0_, *A*_t_, and *A*_e_ are the absorbance values determined at the start of the reaction (*t* = 0 s), at time *t*, and at equilibrium, respectively.

3 $$k_{d}=\frac{1}{t}\ln\frac{A_{o}-A_{e}}{A_{t}-A_{e}}$$

The dissociation kinetics data for all Eu^II^-containing complexes at pH 7 were determined by adopting an electrochemical technique.^21^ Cyclic voltammetry was performed to measure peak currents (*i*_p_) of Eu^II^L/Eu^III^L (L = **1**–**4**) at different time points. The instrumentation was the same as described in the cyclic voltammetry section. Acquisition parameters for the cyclic voltammograms of all complexes are similar to those described in the cyclic voltammetry section with a different number of sweeps (between 1,450 and 1,950 sweeps). The use of many sweeps is consistent with other reported voltammetry studies.^22–24^ Samples were prepared under an atmosphere of N_2_ by dissolving Eu^II^-containing complexes (1 mM) and tetraethylammonium perchlorate (100 mM) in degassed water (pH 7). Solutions were sparged with Ar for 5 min while stirring before measurements were performed. Reduction peak current (*i*_pc_) heights were monitored to derive *k*_d_ using eq 4, in which *k*_d_ is the calculated first-order dissociation rate constant; *t* is time; and *C*_0_ and *C* are the concentration values determined at the start of the dissociation and at the time *t*, respectively.

4 $$\ln\left[C\right]=-k_{d}t+\ln[{C]}_{0}$$

Because the peak current in cyclic voltammetry is proportional to the concentration of analyte, eq 4 can be written as eq 5, in which *i*_p0_ and *i*_p_ are the current peak heights determined at the start of the dissociation and at time *t*, respectively.

5 $$\ln i_{p}=-k_{d}t+\ln i_{p0}$$

Values of *k*_d_ were obtained as the slope of the plot of the natural log of peak current *i*_p_ versus time.

### 2.6 Statistical Analysis

Two-sided unpaired *t*-tests (*p* < 0.05) were used to compare mean (n = 3) *E*_1/2_ values for **Eu** ^**II/III**^ **2** and **Eu** ^**II/III**^ **3** and also for **Eu** ^**II/III**^ **3** and **Eu** ^**II/III**^ **4**. Unpaired *t*-tests were also performed to compare mean (*n* = 3) *k*_d_ values at pH 7 among **Eu** ^**II**^ **1**–**Eu** ^**II**^ **4** and at pH 1 among **Eu** ^**II**^ **2**–**Eu** ^**II**^ **4**.

### 2.7 Calculation of Distance between Metal Ion and Peripheral Charges

For **Eu** ^**II**^ **1**–**Eu** ^**II**^ **4**, the maximum-possible distances between Eu ions and anionic functional groups on the periphery of the ligands were calculated from three-dimensional chemical structures drawn using PerkinElmer Chem3D (version 21.0.0.28) software.

### 2.8 Synthetic Procedures and Characterization

Synthetic routes for **Eu** ^**II**^ **1**, **Eu** ^**II**^ **3**, and **Eu** ^**II**^ **4** are shown in Scheme S1 followed by detailed synthetic procedures.

#### 2.8.1 Europium­(II) 2,2’,2’’,2’’’-(1,4,7,10-tetraazacyclododecane-1,4,7,10-tetrayl)­tetraacetate disodium (**Eu** ^**II**^ **1**)

In a wet glovebox under an atmosphere of N_2_, a solution of EuCl_2_ (27.0 mg, 0.112 mmol, 1.05 equiv) was added to a stirred solution of **1** (55.0 mg, 0.107 mmol, 1.0 equiv) in degassed water (8 mL). The pH of the resulting solution was adjusted to 6.5 with degassed aqueous NaOH (1 M). The reaction mixture was stirred at 65 °C for 24 h at which point the pH was adjusted to 9 using aqueous NaOH (1 M) to precipitate excess europium. The resulting yellow solution was filtered three times through polytetrafluoroethylene syringe filters. The pH of the resulting solution was adjusted to 7 using degassed aqueous HCl (1 M). Solutions of **Eu** ^**II**^ **1** were characterized with UV–visible spectroscopy and luminescence spectroscopy to confirm the formation of **Eu** ^**II**^ **1** (Figure S1). The concentration of solutions of **Eu** ^**II**^ **1** was determined with ED-XRF spectroscopy.

#### 2.8.2 2,2’,2’’,2’’’-((2,2’,2’’,2’’’-((2,2’,2’’,2’’’-(1,4,7,10-Tetraazacyclododecane-1,4,7,10-tetrayl)­tetrakis­(acetyl))­tetrakis­(azanediyl))­tetrakis­(acetyl))­tetrakis­(azanediyl))­tetraacetic acid (**3**)

A stirring mixture of **5** (5.013 g, 21.18 mmol), K_2_CO_3_ (5.180 g, 37.48 mmol), and cyclen (0.803 g, 4.66 mmol) in acetonitrile (210 mL) was heated at 90 °C for 72 h. The mixture was filtered while hot through Celite, and the filtrate was concentrated under reduced pressure to yield a sticky brown solid. The solid was dissolved in CH_2_Cl_2_ (80 mL), and the resulting solution was filtered using filter paper. The filtrate was dried over sodium sulfate and then filtered onto filter paper. Solvent was removed from the filtrate under reduced pressure to yield a light-brown solid that was dissolved in aqueous HCl (6 M, 156 mL), and the resulting solution was stirred for 18 h. The resulting solution was filtered through filter paper, and water was removed under reduced pressure to yield **3** as a white solid (1.843 g, 72%). HRMS (*m*/*z*): [M – H]^−^ calcd. for C_32_H_51_N_12_O_16_, 859.3511; found, 859.3562; Anal. Calcd for C_32_H_49_N_12_Na_3_O_16_·4.55H_2_O: C, 38.10; H, 5.81; N, 16.66. Found: C, 38.58; H, 5.85; N, 16.17. Thermogravimetric analysis (TGA) confirmed the presence of associated water molecules (Figure S14).

#### 2.8.3 Europium­(III) 2,2’,2’’,2’’’-((2,2’,2’’,2’’’-((2,2’,2’’,2’’’-(1,4,7,10-tetraazacyclododecane-1,4,7,10-tetrayl)­tetrakis­(acetyl))­tetrakis­(azanediyl))­tetrakis­(acetyl))­tetrakis­(azanediyl))­tetraacetate sodium (**Eu** ^**III**^ **3**)

A solution of EuCl_3_·6H_2_O (0.965 g, 2.63 mmol, 1.1 equiv) was added to a stirred solution of **3** (2.06 g, 2.39 mmol, 1.0 equiv) in water (18 mL). The pH of the resulting solution was adjusted to 6.5 with aqueous NaOH (1 M). The reaction mixture was stirred at 65 °C for 24 h at which point the pH was adjusted to 9 using aqueous NaOH (1 M) to precipitate excess europium. Centrifugation of the resulting mixture was followed by decanting and filtering through polytetrafluoroethylene syringe filters. The pH of the resulting solution was adjusted to 7 using aqueous HCl (1 M). The solution was concentrated under reduced pressure to obtain a sticky white solid that was dissolved in minimum amount of water and desalted using PD MidiTrap G-10 desalting size exclusion columns (Cytiva) until no Cl^–^ was detected by elemental analysis. The desalted samples were freeze-dried to obtain 0.405 g (16%) of **Eu** ^**III**^ **3** as a white solid. HRMS (*m*/*z*): [M]^−^ calcd. for C_32_H_48_EuN_12_O_16_, 1009.2529; found, 1009.2566; Anal. Calcd for C_32_H_48_EuN_12_Na_1.92_O_16_·6.7H_2_O: C, 32.75; H, 5.27; N, 14.32. Found: C, 32.25; H, 4.67; N, 13.90. TGA confirmed the presence of associated water molecules (Figure S14).

#### 2.8.4 Europium­(II) 2,2’,2’’,2’’’-((2,2’,2’’,2’’’-((2,2’,2’’,2’’’-(1,4,7,10-tetraazacyclododecane-1,4,7,10-tetrayl)­tetrakis­(acetyl))­tetrakis­(azanediyl))­tetrakis­(acetyl))­tetrakis­(azanediyl))­tetraacetate disodium (**Eu** ^**II**^ **3**)

In a wet glovebox under an atmosphere of N_2_, **Eu** ^**II**^ **3** was prepared by stirring of **Eu** ^**III**^ **3** (162.7 mg, 140.0 μmol, 1.0 equiv) with Zn dust (1.657 g, 25.34 mmol, 181 equiv) in degassed water (10 mL) for 2 h at ambient temperature. The resulting yellow supernatant was filtered through a polytetrafluoroethylene syringe filter, and the yellow filtrate was swirled with DOWEX-Na^+^ (0.15 g) for 2 min followed by filtration using a polytetrafluoroethylene syringe filter. The DOWEX cation-exchange step was repeated a total of three times to yield a bright-yellow solution of **Eu** ^**II**^ **3**. Solutions of **Eu** ^**II**^ **3** were characterized with UV–visible spectroscopy to confirm the reduction of Eu^III^ to Eu^II^ and luminescence spectroscopy to confirm loss of Eu^III^ (Figure S2). The concentration of **Eu** ^**II**^ **3** in each solution was determined with energy-dispersive X-ray fluorescence (ED-XRF) spectroscopy.

#### 2.8.5 Ethyl (2-chloroacetyl)­glycylglycylglycinate (**6**)

A solution of ethyl glycylglycineglycinate hydrochloride (5.309 g, 20.93 mmol) in dimethylformamide (175 mL) at 0 °C was treated with chloroacetyl chloride (5.1 mL, 64 mmol) dropwise for 15 min. The reaction mixture was removed from the ice bath and stirred for 1.5 h at ambient temperature and then concentrated under reduced pressure. The solid was washed with CH_2_Cl_2_ (400 mL) to yield 5.890 g (96%) of **6** as a glassy white solid. ^1^H NMR (500 MHz, (CD_3_)_2_SO): δ = 1.20 (t, *J* = 7.1, 3H), 3.76 (d, *J* = 5.9, 2H), 3.80 (d, *J* = 5.7, 2H), 3.83 (d, *J* = 5.9, 2H), 4.10 (q, *J* = 7.1, 2H), 4.14 (s, 2H), 8.26 (td, *J* = 2.3, 5.9, 2H), 8.42 (t, *J* = 5.6, 1H); ^13^C NMR (126 MHz, (CD_3_)_2_SO): δ 15.0, 41.5, 42.6, 43.2, 43.5, 61.4, 167.2, 169.4, 170.2, 170.6; HRMS (*m*/*z*): [M + H]^+^ calcd. for C_10_H_17_ClN_3_O_5_, 294.0851; found, 294.0848.

#### 2.8.6 2,2’,2’’,2’’’-((2,2’,2’’,2’’’-((2,2’,2’’,2’’’-((2,2’,2’’,2’’’-(1,4,7,10-Tetraazacyclododecane-1,4,7,10-tetrayl)­tetrakis­(acetyl))­tetrakis­(azanediyl))­tetrakis­(acetyl))­tetrakis­(azanediyl))­tetrakis­(acetyl))­tetrakis­(azanediyl))­tetraacetic acid (**4**)

A stirring mixture of **6** (4.312 g, 14.68 mmol), K_2_CO_3_ (3.529 g, 25.54 mmol), cyclen (0.550 g, 3.19 mmol), and acetonitrile (190 mL) was heated at 90 °C for 72 h. The mixture was filtered while hot through Celite, and the filtrate was concentrated under reduced pressure to yield a sticky brown solid. The solid was dissolved in methanol (50 mL), and the resulting solution was filtered using filter paper. The filtrate was dried over sodium sulfate and then filtered through filter paper. Solvent was removed from the filtrate under reduced pressure to yield a light-brown solid that was dissolved in aqueous HCl (6 M, 150 mL), and the resulting solution was stirred for 18 h. The solution was filtered through a filter paper, and water was removed under reduced pressure to yield 1.063 g (31%) of **4** as a white solid. HRMS (*m*/*z*): [M – H]^−^ calcd. for C_40_H_63_N_16_O_20_, 1087.4410; found, 1087.4412; Anal. Calcd for C_42_H_69_N_16_Na_3_O_22_·4H_2_O: C, 39.07; H, 6.01; N, 17.36. Found: C, 38.97; H, 5.68; N, 16.87. TGA confirmed the presence of associated water molecules (Figure S14).

#### 2.8.7 Europium­(III) 2,2’,2’’,2’’’-((2,2’,2’’,2’’’-((2,2’,2’’,2’’’-((2,2’,2’’,2’’’-(1,4,7,10-tetraazacyclododecane-1,4,7,10-tetrayl)­tetrakis­(acetyl))­tetrakis­(azanediyl))­tetrakis­(acetyl))­tetrakis­(azanediyl))­tetrakis­(acetyl))­tetrakis­(azanediyl))­tetraacetate sodium (**Eu** ^**III**^ **4**)

An aqueous solution of (3 mL) EuCl_3_·6H_2_O (0.354 g, 0.966 mmol, 1.1 equiv) was added to a stirred solution of **4** (0.956 g, 0.878 mmol, 1.0 equiv) in water (13 mL). The pH of the resulting solution was adjusted to 6.5 with aqueous NaOH (1 M). The reaction mixture was stirred at 65 °C for 48 h. Then, the pH was adjusted to 9 using aqueous NaOH (1 M) to precipitate excess europium. Centrifugation of the resulting mixture was followed by decanting and filtering through polytetrafluoroethylene syringe filters. The pH of the resulting solution was adjusted to 7 using aqueous HCl (1 M). The solution was concentrated under reduced pressure to obtain a sticky white solid that was dissolved in water and desalted using PD MidiTrap G-10 desalting size exclusion columns (Cytiva) until no Cl^–^ was detected by elemental analysis. The desalted samples were freeze-dried to obtain 0.186 g (17%) of **Eu** ^**III**^ **4** as a white solid. HRMS (*m*/*z*): [M]^−^ calcd. for C_32_H_60_EuN_16_O_20_, 1237.3404; found, 1237.3400; Anal. Calcd for C_40_H_60_EuN_16_Na_1.97_O_20_·7.25H_2_O: C, 33.16; H, 5.46; N, 15.47. Found: C, 33.13; H, 5.39; N, 14.97. TGA confirmed the presence of associated water molecules (Figure S14).

#### 2.8.8 Europium­(II) 2,2’,2’’,2’’’-((2,2’,2’’,2’’’-((2,2’,2’’,2’’’-((2,2’,2’’,2’’’-(1,4,7,10-tetraazacyclododecane-1,4,7,10-tetrayl)­tetrakis­(acetyl))­tetrakis­(azanediyl))­tetrakis­(acetyl))­tetrakis­(azanediyl))­tetrakis­(acetyl))­tetrakis­(azanediyl))­tetraacetate disodium (Eu^II^4)

In a wet glovebox under an atmosphere of N_2_, **Eu** ^**II**^ **4** was prepared by stirring **Eu** ^**III**^ **4** (201.8 mg, 140.0 μmol, 1 equiv) with Zn^0^ dust (1.657 g, 25.34 mmol, 181 equiv) in degassed water (10 mL) for 2 h at ambient temperature. The resulting yellow supernatant was filtered through polytetrafluoroethylene syringe filters, and the yellow filtrate was swirled with DOWEX-Na^+^ (0.15 g) for 2 min followed by filtration using a polytetrafluoroethylene syringe filter. The DOWEX cation-exchange step was repeated a total of three times to yield a bright yellow solution of **Eu** ^**II**^ **4**. Solutions of **Eu** ^**II**^ **4** were characterized with UV–visible spectroscopy to confirm the reduction of Eu^III^ to Eu^II^ and luminescence spectroscopy to confirm loss of Eu^III^ (Figure S3). The concentration of solutions of **Eu** ^**II**^ **4** was determined with ED-XRF spectroscopy.

## 3 Results and Discussion

To test our hypotheses, we synthesized **Eu** ^**II**^ **1–Eu** ^**II**^ **4**. Although phosphonates are used in lanthanide coordination chemistry because they provide strong metal–ligand interactions,^25–27^ multidentate phosphonate-based systems are synthetically challenging compared to systems containing other anions like carboxylates. Additionally, based on protonation state, carboxylate groups only have charges of 0 or −1, whereas phosphonates have charges of 0, −1, or −2. Therefore, using carboxylates greatly reduces the number of possible complexities in solution, enabling more facile interpretation of results. For these reasons, we selected carboxylate groups to systematically probe the influence of peripheral anionic functionalities on the properties of complexes of Eu^II^. To incorporate carboxylates in a systematic manner, we started with known systems: the Eu^II^-containing complex of 1,4,7,10-tetraazacyclododecane-1,4,7,10-tetraacetic acid (DOTA), **Eu** ^**II**^ **1**, provides a parent reference, and the tetraglycinate analogue (**Eu** ^**II**^ **2**) extends the peripheral anionic charge outward from Eu^II^ through the incorporation of one glycine unit. Further, both complexes have been reported with Eu^II^,^10,28^ providing a solid foundation upon which to build our studies. Building on this foundation, we designed and synthesized **Eu** ^**II**^ **3** and **Eu** ^**II**^ **4**, in which additional glycine spacers were introduced to increase the distance between Eu^II^ and anionic functional groups on the periphery of the ligands. Because the series **Eu** ^**II**^ **1**–**Eu** ^**II**^ **4** varies the distance between Eu and peripheral carboxylic acids with glycine spacers, only modest changes in the acidity of the carboxylic acids are expected, consistent with reported p*K*_a_ values for glycine (p*K*_a_ = 2.33), diglycine (p*K*_a_ = 3.19), and triglycine (p*K*_a_ = 3.19).^29^ At neutral pH, all peripheral carboxylates are expected to be essentially fully deprotonated, rendering such small p*K*_a_ differences inconsequential. This stepwise extension of pendant arms provided a controlled way to test our hypotheses involving the influence of the distance of peripheral ligand charges and Eu^II^ on electrochemical potentials and dissociation rates. In interpreting the distance–stabilities correlations, we note that the use of maximum-possible distances rather than average Eu–anionic functional group distances reflects the conformational flexibility of pendant arms. In solution, the arms undergo free rotation about many bonds resulting in a dynamic range of distances. Because the instantaneous or average Eu–peripheral anionic charge distance is not easily defined, we chose to look at maximum-possible distance that can be calculated. Additionally, the trends for either average or maximum-possible distance would be expected to be the same because the average distance is expected to increase proportionally to the maximum-possible distance. Although this approximation does not capture dynamic fluctuations, it enables meaningful comparisons for correlating ligand design with observed electrochemical and kinetic trends.

**Eu** ^**II**^ **1** was synthesized by metalating the commercially available ligand **1** with EuCl_2_. The resulting yellow solution was characterized with UV–visible spectroscopy to confirm the formation of **Eu** ^**II**^ **1** (Figure S1). To probe for the absence of Eu^III^ in the yellow solution, luminescence spectroscopy and minimum detectable concentration experiments were conducted (Figures S1 and S6). Minimum detectable concentration values were determined to be 2.4 μM for **Eu** ^**III**^ **1**. The emission spectra of **Eu** ^**III**^ **1** contained the characteristic emission bands from the radiative decay of the ^5^D_0_ excited state of Eu^III^ to the ^7^F manifold, but the yellow solution of **Eu** ^**II**^ **1** did not produce observable Eu^III^-based emissions despite having the same concentration of Eu. **Eu** ^**II**^ **2** was prepared following reported procedure,^10^ and characterization matched reported values. To prepare the new complexes, ligands **3** and **4** were synthesized in two steps from commercially available starting materials and metalated with EuCl_3_ to produce **Eu** ^**III**^ **3** and **Eu** ^**III**^ **4**. Elemental analyses confirmed purity and high-resolution mass spectrometry confirmed the identity of the complexes. **Eu** ^**II**^ **3** and **Eu** ^**II**^ **4** were synthesized by reducing **Eu** ^**III**^ **3** and **Eu** ^**III**^ **4** with Zn^0^. Yellow solutions of **Eu** ^**II**^ **3** and **Eu** ^**II**^ **4** were characterized with UV–visible spectroscopy to confirm the reduction of Eu^III^ to Eu^II^ (Figures S2 and S3). To probe for the absence of Eu^III^ in the yellow solutions, luminescence spectroscopy and minimum detectable concentration experiments were conducted (Figures S2, S3, and S6). Minimum detectable concentration values were determined to be 1.5 and 2.1 μM for **Eu** ^**III**^ **3** and **Eu** ^**III**^ **4**, respectively. The emission spectra of both **Eu** ^**III**^ **3** and **Eu** ^**III**^ **4** contained the characteristic emission bands from the radiative decay of the ^5^D_0_ excited state of Eu^III^ to the ^7^F manifold, but the yellow solutions of **Eu** ^**II**^ **3** and **Eu** ^**II**^ **4** did not produce observable Eu^III^-based emissions despite having the same concentration of Eu (Figures S2 and S3). The absence of Eu^**III**^ emission in solutions of **Eu** ^**II**^ **3** and **Eu** ^**II**^ **4** combined with the minimum detectable concentrations of **Eu** ^**III**^ **3** and **Eu** ^**III**^ **4** indicate that the reduction reaction reduced ≥ 99.0% of the Eu^III^. The data suggest that treatment with Zn^0^ is responsible for the lack of Eu^III^-based emissions in the yellow solution, and that the species imparting the yellow color is oxidized by air to regenerate Eu^III^-containing complexes. This observation is in reasonable agreement with data published for synthesis of other divalent europium complexes.^4,10^ Additionally, the emission spectra of **Eu** ^**III**^ **1**, **Eu** ^**III**^ **2**,^10^ **Eu** ^**III**^ **3**, and **Eu** ^**III**^ **4** contain similar spectral features and relative intensities, indicating comparable Eu^III^ coordination environments in solution, supporting the hypothesis that differences among the complexes primarily arise from peripheral structural variation rather than changes in inner-sphere geometry. The UV–visible molar extinction coefficient spectra showed broad absorptions at pH 1 for **Eu** ^**II**^ **3** at 356 nm (molar extinction coefficient, ε = 9.24 × 10^2^ M^–1^ cm^–1^) and for **Eu** ^**II**^ **4** at 357 nm (ε = 8.81 × 10^2^ M^–1^ cm^–1^). These absorptions are consistent with 4f–5d electronic transitions characteristic of Eu^II^.^30^

Electrochemical potentials of the Eu^II/III^ couple are critical parameters in the design of Eu^II^-containing probes. To explore the dependence of electrochemical potentials (*E*_1/2_) on the distances between metal ions and anionic functional groups on the periphery of the ligands, trends of redox couples **Eu** ^**II/III**^ **1**, **Eu** ^**II/III**^ **2**, **Eu** ^**II/III**^ **3**, and **Eu** ^**II/III**^ **4** were studied. Cyclic voltammetry was conducted to characterize the electrochemical potential of europium complexes, resulting in reversible one-electron redox couples with *E*_1/2_ values of −1.142 ± 0.005, −0.964 ± 0.001, −0.952 ± 0.002, and −0.938 ± 0.003 V versus Ag/AgCl for redox couples of **Eu** ^**II/III**^ **1**, **Eu** ^**II/III**^ **2**, **Eu** ^**II/III**^ **3**, and **Eu** ^**II/III**^ **4**, respectively (Figures 2, S7, and Table S1). We compared the mean (n = 3) *E*_1/2_ values between **Eu** ^**II/III**^ **2** and **Eu** ^**II/III**^ **3** and between **Eu** ^**II/III**^ **3** and **Eu** ^**II/III**^ **4** using unpaired *t*-tests (*p* < 0.05 for both comparisons), and the *E*_1/2_ values were different from each other in each comparison (Table S2).

**Figure 2:**
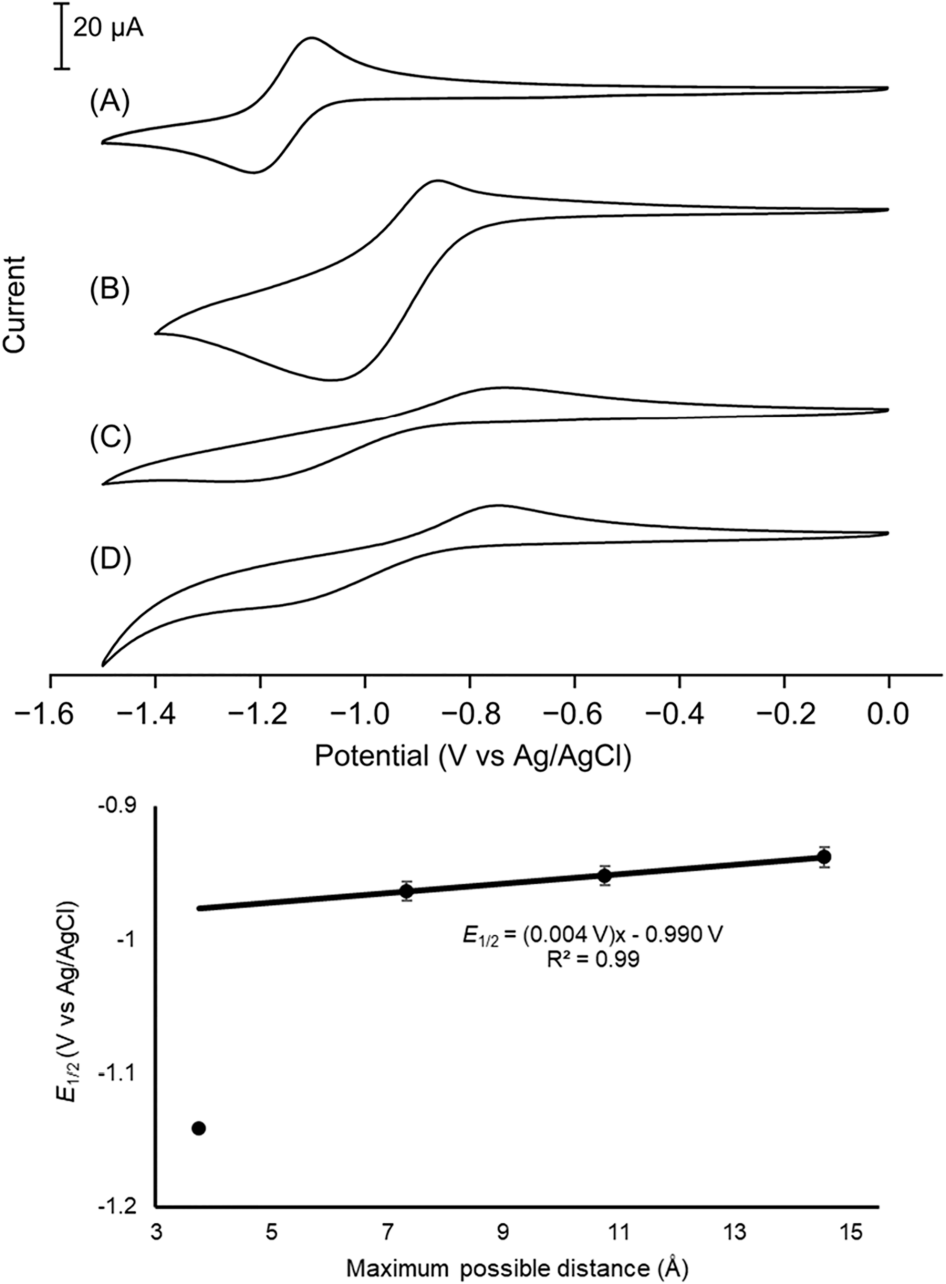
(Top) Cyclic voltammograms of (A) **Eu** ^**II/III**^ **1**, (B) **Eu** ^**II/III**^ **2**, (C) **Eu** ^**II/III**^ **3**, and (D) **Eu** ^**II/III**^ **4** (3 mM) with tetraethylammonium perchlorate (50 mM) as the supporting electrolyte at pH 7. The scale bar on the *y*-axis applies to all cyclic voltammograms in the figure. (Bottom) Plot showing the relationship of *E*_1/2_ versus the calculated maximum-possible distances between metal ions and anionic functional groups on the periphery of the ligands. The best-fit trendline was drawn using data from **Eu** ^**II/III**^ **2**, **Eu** ^**II/III**^ **3**, and **Eu** ^**II/III**^ **4**.

In our experiments, cyclic voltammetry highlights the role of ligand architecture on electrochemical potential. We sought to compare the Eu^II/III^ couple of Eu-containing complexes of **1**–**4** that differ in the maximum-possible distances between Eu^II^ and peripheral ligand charges (Figure 1). Ligands change the redox properties of Eu ions, and the redox behavior of **Eu** ^**II/III**^ **1** (−1.142 V versus Ag/AgCl) and **Eu** ^**II/III**^ **2** (−0.964 V versus Ag/AgCl) are in reasonable agreement with reported data for these complexes.^28,31^ As the maximum-possible distances between Eu^II^ ions and peripheral anionic functional groups increased beyond **Eu** ^**II/III**^ **2**, more positive *E*_1/2_ values of **Eu** ^**II/III**^ **3** (−0.952 V versus Ag/AgCl) and **Eu** ^**II/III**^ **4** (−0.938 V versus Ag/AgCl) were observed. This trend is likely due to lowering in free energy of Eu^II^ as a function of increasing maximum-possible peripheral charge distances, making the divalent state more favorable relative to its Eu^III^ analogue: briefly, anionic groups near Eu^II^ raise its free energy through electrostatic repulsion, making oxidation (Eu^II^ → Eu^III^ + e^–^) thermodynamically more favorable. As the negative charges are positioned farther from the metal ion, Eu^II^ becomes relatively more stable, requiring a greater driving force for oxidation, reflected in more positive potentials. The entire Eu^II/III^ redox group shows the same trend, but the *E*_1/2_ gap (178 mV) between **Eu** ^**II/III**^ **1** and **Eu** ^**II/III**^ **2** is substantially longer than the other gaps. The shift in *E*_1/2_ between **Eu** ^**II/III**^ **1** and **Eu** ^**II/III**^ **2** is consistent with electrochemical studies of Eu^II/III^-containing DOTA-based complexes, where replacing four carboxylates with four amide donors changes *E*_1/2_ by 168 mV.^31^ The direct anionic coordination in **Eu** ^**II/III**^ **1** increases electron density around the electron-rich Eu^II^ ion, making oxidation more likely and shifting *E*_1/2_ to more negative potentials. Therefore, the data suggest that donor identity has a greater influence on redox potential than distance of peripheral charge. Because **Eu** ^**II/III**^ **2**–**Eu** ^**II/III**^ **4** have the same donor set, we were able to study the influence of peripheral charge distance using these three complexes.

*E*_1/2_ values of **Eu** ^**II/III**^ **2**–**Eu** ^**II/III**^ **4** were plotted against the maximum-possible distances between Eu^II^ ions and peripheral anionic groups, yielding a linear fit that defines the relationship between electrochemical potential and charge separation (Figure 2). Because electrostatic potential scales as 1/distance, we present *E*_1/2_ as a function of distance. The linear regression model describing the relationship between *E*_1/2_ and metal–peripheral ligand charge separation fits well with the data for **Eu** ^**II/III**^ **2**, **Eu** ^**II/III**^ **3**, and **Eu** ^**II/III**^ **4**. From the plot in Figure 2, we calculated a positive shift of *E*_1/2_ of 4 mV per 3.6 Å increase in maximum-possible distance between peripheral charge and Eu. To contextualize the 4 mV per 3.6 Å shift observed in our study, we compared it with *E*_1/2_ changes from other reported structural modifications of europium-containing complexes. Sequential replacement of carboxylate with amide donors in DOTA-based Eu^II^ complexes produces 42 mV shifts per substitution,^31^ and picolinate-to-picolinamide pendant arm substitutions in Eu^II^-containing complexes of 1,10-diaza-18-crown-6 derivatives systems yield ∼150 mV per change.^31,32^ Expanding the macrocyclic ring size from 12-membered to 14-membered 1,4,8,11-tetraazacyclotetradecane-1,4,8,11-tetraacetic acid in Eu^II^-containing complexes produces a 139 mV shift, attributed to the smaller ring favoring the smaller Eu^III^ ion whereas the larger ring better accommodates Eu^II^.^28^ Switching to Eu^II^ complexes of 18-membered macrocycles generates large positive shifts of 215–315 mV relative to DOTA,^9,28^ whereas the noncyclic Eu^II^ diethylenetriamine-*N*,*N′*,*N″*,*N‴*,*N‴′*-pentaacetate shows a 530 mV difference from 1,4,10,13-tetraoxa-7,16-diaza-cyclooctadecane-7,16-diacetate.^9^ Therefore, our observed shift of 4 mV per 3.6 Å is 10–130 times smaller than these other structural changes, corroborating that modulation of *E*_1/2_ through spatial positioning of peripheral charges represents a fine-tuning mechanism as opposed to coarse tuning associated with the other changes. Overall, the *E*_1/2_ data collected using the europium-containing complexes in this study indicates that the longer maximum-possible distance between Eu^II^ ions and anionic functional groups on the periphery of ligands thermodynamically favors divalent europium relative to the shorter one, but only to a small extent relative to other structural changes made at the metal ion.

Beyond electrochemical potential, the inertness of Ln-containing complexes from dissociation of metal ions is an essential parameter for their use as contrast agents because insufficient inertness is associated with release of Eu^II^ that is readily oxidized resulting in a stop in contrast enhancement and has potential toxicity from uncomplexed lanthanide ions. We anticipated that because of the nature of electrostatic interactions, the possibility exists to influence metal dissociation via control of the distance between Eu^II^ ion and charges on ligands. To study the dissociation of Eu^II^-containing complexes as a function of the influence of the distance between and Eu ions and anionic functional groups on the periphery of the ligands in complexes **Eu** ^**II**^ **1**–**Eu** ^**II**^ **4**, we determined rates of dissociation using an electrochemical technique to monitor the dissociation of Eu^II^ complexes at pH 7. In the experiment, peak heights of complexed Eu^II^ were used to measure peak current of Eu^II^ at different time points, and the data were plotted against time (Table 1 and Figures S8–S11). We observed that with increasing of the maximum-possible distances between Eu ions and anionic functional groups on the periphery of the ligands, dissociation rates slowed. Statistical significance was assessed for pH 7 data using unpaired *t*-test and found *p* = 0.04 for **Eu** ^**II**^ **1** versus **Eu** ^**II**^ **4**, and *p* = 0.02 for **Eu** ^**II**^ **2** versus **Eu** ^**II**^ **4** (Table S3). At pH 7, the dissociation of Eu^II^-containing complexes is not dominated by acid-assisted pathways but instead reflects the intrinsic kinetic stability imparted by ligand architecture. The *k*_d_ values of **Eu^II^1**–**Eu^II^4** at pH 7 (Table 1) revealed a clear order of stability: **Eu^II^1** dissociated fastest (4.5 × 10^–5^ s^–1^) followed by **Eu** ^**II**^ **2** (2.8 × 10^–5^ s^–1^) and **Eu** ^**II**^ **3** (1.7 × 10^–5^ s^–1^), with **Eu^II^4** dissociating the slowest (1.1 × 10^–5^ s^–1^). The differences were significant for **Eu** ^**II**^ **1** versus **Eu** ^**II**^ **4** and **Eu** ^**II**^ **2** versus **Eu** ^**II**^ **4**. **Eu** ^**II**^ **1** is the fastest but is not included in the remainder of comparisons in this section because the carboxylic acid groups in **1** directly coordinate Eu^II^, unlike with **2**–**4**. In comparison to **Eu^II^2**, **Eu^II^3** and **Eu^II^4** show 1.5–2.5-fold slower dissociations. **Eu^II^2** and **Eu^II^4** show a stepwise slowing of dissociation (*p* = 0.02) as a function of increasing of the maximum-possible distance between Eu**^II^** and peripheral anionic functional groups (Tables 1 and S3). That result suggests that glycinamide pendant-arm length contributes modestly to enhanced inertness of the complexes.

**Table 1:** *k*_d_ Values at pH 1 and 7

	pH 1	pH 7
complex	*k*_d_ (×10^–4^ s^–1^)^a^	*k*_d_ (×10^–5^ s^–1^)^a^
**Eu** ^**II**^ **1**	277 ± 41	4.5 ± 0.9
**Eu** ^**II**^ **2**	13.2 ± 0.3	2.8 ± 0.3
**Eu** ^**II**^ **3**	12.5 ± 0.3	1.7 ± 0.2
**Eu** ^**II**^ **4**	11.9 ± 0.1	1.1 ± 0.2

a *k*_d_ values represent mean ± standard error (*n* = 3).

To contextualize the 1.5–2.5-fold variation observed in our study, we compared it with *k*_d_ changes from other reported structural modifications. Addition of a benzo group to Eu**^II^** cryptand frameworks increases dissociation rates by 4.2-fold, while incorporation of an electron-withdrawing fluorine substituent produces an 8.5-fold increase relative to the unsubstituted cryptand.^33^ Ring-size modifications produce dramatically larger effects: expanding the macrocyclic ring by replacing a carboxylate arm with a propionate from 12-membered Gd-DOTA to 13-membered Gd-(1,4,7,10-tetraazacyclotridecane-1,4,7,10-tetrayl)­tetraacetic acid (Gd-TRITA) results in an 840-fold decrease in dissociation rate.^34^ Complete ligand rigidification through cross-bridging in 1,4,8,11-tetraazacyclotetradecane (cyclam)-derived lanthanide­(III)-containing complexes results in no dissociation for at least five months,^35^ 18-membered pyridine-rigidified lanthanide­(III)-containing complexes with coordination number ten are 100–10,000-fold more inert than that of the Ln-DOTA due to three-dimensional wrapping of metal ions inside the ligand cavity.^36^ Therefore, our observed shift of 1.5–2.5-fold in *k*_d_ corroborating that modulation of *k*_d_ through spatial positioning of peripheral charges represents a fine-tuning mechanism as opposed to coarse tuning associated with the other structural changes on ligands. The slowed dissociation with increasing of the maximum-possible distances between Eu^II^ ions and peripheral anionic functional groups can be rationalized by factors beyond Coulombic interactions.

First, macrocyclic and steric effects are known to influence dissociation kinetics of lanthanide­(III)-containing complexes.^37,38^ Ligand bulk reduces the accessibility of the metal ion to the environment, thereby enhancing kinetic inertness. This effect is particularly pronounced in rigid ligands that result in three-dimensional coordination environments that shield metal ions and confer resistance from decomplexation.^35^ In **Eu** ^**II**^ **1**–**Eu** ^**II**^ **4**, the extended pendant arms likely provide additional steric shielding, which decreases the probability of competing donors reaching Eu^II^. This effect slows dissociation despite the weaker direct Coulombic stabilization. Second, intramolecular interactions among the pendant arms generate secondary stabilization. In systems containing peptide-like groups, noncovalent forces such as hydrogen bonding and hydrophobic interactions are well documented to drive self-assembly.^39,40^ These self-assembled systems could be envisioned to reduce solvent exposure of a coordinated metal ion. A study highlighted the role of hydrogen bonding in Eu^III^-DOTA-tetraamide complexes, showing that intramolecular hydrogen-bonding correlates with slow water-exchange kinetics.^41^ Additionally, Eu^III^-containing complexes of 1,4,7,10-tetraazacyclododecane-1,4,7-triacetic acid-derived ligands exhibit competitive intramolecular carboxylate coordination,^42^ evidencing pendant groups folding back to participate in coordination. By analogy, the longer arms in **Eu** ^**II**^ **3** and **Eu** ^**II**^ **4** might fold back or interact intramolecularly, producing a more stabilized coordination environment around Eu^II^, thereby slowing dissociation relative to **Eu** ^**II**^ **2**. Together the increased steric protection and ligand self-interactions outweigh the weakening of electrostatic interactions at pH 7 in Eu^II^-containing macrocyclic system functionalized with peptide-based pendant arms. Therefore, the trend of increasing distances between Eu ions and peripheral anionic groups in **Eu** ^**II**^ **1**–**Eu** ^**II**^ **4** is associated with fine-tuned decreasing dissociation rates.

As a control experiment for studying the effect of pendent charge on dissociation, we measured the dissociation data for all complexes at pH 1 where all the carboxylates are protonated, and thus not charged. In these experiments, we monitored dissociation using spectrophotometry at 420 nm (Table 1 and Figures S12–S13). The absorbance at 420 nm was selected because it is within an absorbance range specific to Eu^II^-containing complexes, whereas the product of dissociation, the Eu^II^ aqua ion, does not absorb beyond 412 nm (Figure S12). We found a rapid dissociation in the case of **Eu** ^**II**^ **1**, but **Eu** ^**II**^ **2**–**Eu** ^**II**^ **4** did not have values of *k*_d_ that were different from each other at pH 1 (Tables 1 and S4). The measured value for **Eu** ^**II**^ **2** was consistent with a reported value.^10^ The rapid dissociation of **Eu** ^**II**^ **1** at pH 1 (277 × 10^–4^ s^–1^) arises from the protonation of the carboxylate functional groups directly coordinated to Eu. Protonation of carboxylates to form carboxylic acids directly influences coordination by weakening Eu–O interactions. For **Eu^II^2**, **Eu^II^3**, and **Eu^II^4**, the *k*_d_ values at pH 1 (11.9 × 10^–4^ to 13.2 × 10^–4^ s^–1^) are not significantly different from each other (Tables 1 and S4) because all four carboxylates were protonated and, thus, not anionic. Additionally, the *k*_d_ values of **Eu^II^2**–**Eu^II^4** complexes are 47–108-fold slower at pH 7 relative to pH 1. These differences observed with Eu^II^ are similar to the trend observed with Eu^III^: the *k*_d_ of **Eu^III^2** is slower in basic compared to acidic conditions.^43^

Viewed alongside the electrochemical data, a consistent picture emerges in which increasing the distance between Eu^II^ ions and peripheral anionic functional groups shifts the *E*_1/2_ to slightly more positive values and slows dissociation. This duality underlines the importance of ligand architecture, where the spatial arrangement of anionic functional groups on the ligand periphery enables fine-tuning to balance redox potentials and kinetic inertness, although structural changes play a dominant role in controlling *E*_1/2_ and kinetic inertness. Therefore, incorporation of charge on the periphery of ligands to kinetically influence oxidation is not likely to cause substantial changes in *E*_1/2_ and dissociation behavior. However, such design principles could offer a rational approach for ligand design strategies aimed at developing Eu-based contrast agents for hypoxia imaging applications.

## 4 Conclusions

In summary, we synthesized and characterized a series of four Eu^II^-containing macrocyclic complexes in which the maximum-possible distance between the Eu^II^ and anionic functional groups on the periphery of ligands systematically differed across the series. Electrochemical studies of the complexes showed that increasing the distance between the Eu^II^ and peripheral anionic charge shifted *E*_1/2_ to slightly more positive values and that dissociation slowed with increasing arm length. Placing charges on the periphery of ligands provides advantages associated with those charges in the second sphere without causing major changes to the coordination chemistry of the inner sphere. The variations in *E*_1/2_ (4 mV per 3.6 Å) and *k*_d_ (1.5–2.5-fold) reported here represent fine-tuning of electrochemical and kinetic properties, in comparison to the coarse-scale changes observed from other ligand changes. These results emphasize the role of charge separation on ligand architecture for fine-tuning of electrochemical potential and kinetic inertness. We expect that such understanding will aid the rational design of Eu^II^-based redox-responsive MRI contrast agents with peripheral charges incorporated to control the second-sphere environment toward increasing the persistence of Eu^II^ in oxygenated environments.

## Supplementary Material



## Notes

Experimental procedures, absorption and emission studies, NMR spectra, minimum detectable concentration study, cyclic voltammograms for electrochemical study, cyclic voltammograms for dissociation studies at pH 7, absorption data for dissociation studies at pH 1, thermogravimetric analyses, and statistical analyses (PDF)
