# Supplementary material for: Controlling the Kinetic and Electrochemical Properties of EuII–Containing Complexes Using Peripheral Charges
Source: Inorg Chem. 2026 Jan 24;65(5):3052–60. doi: 10.1021/acs.inorgchem.5c05456 (PMC12834489; doi:10.1021/acs.inorgchem.5c05456)
Supplement: Supplementary file 1 [file ic5c05456_si_001.pdf]

Controlling the Kinetic and Electrochemical Properties of Eu^{II}-Containing Complexes Using Peripheral Charges

Md Sydul Islam and Matthew J. Allen*

Department of Chemistry, Wayne State University, 5101 Cass Avenue, Detroit, Michigan 48202, USA

*Email: mallen@wayne.edu

**Content**	**Page**
Table of Contents	S1
Experimental procedures	S2
Absorption and emission studies	S3
NMR spectra	S4
Minimum detectable concentration study	S5
Cyclic voltammograms for electrochemical study	S6
Cyclic voltammograms for dissociation studies at pH 7	S8
Absorption data for dissociation studies at pH 1	S12
Thermogravimetric analyses	S13
Statistical analyses	S14

Experimental Procedures

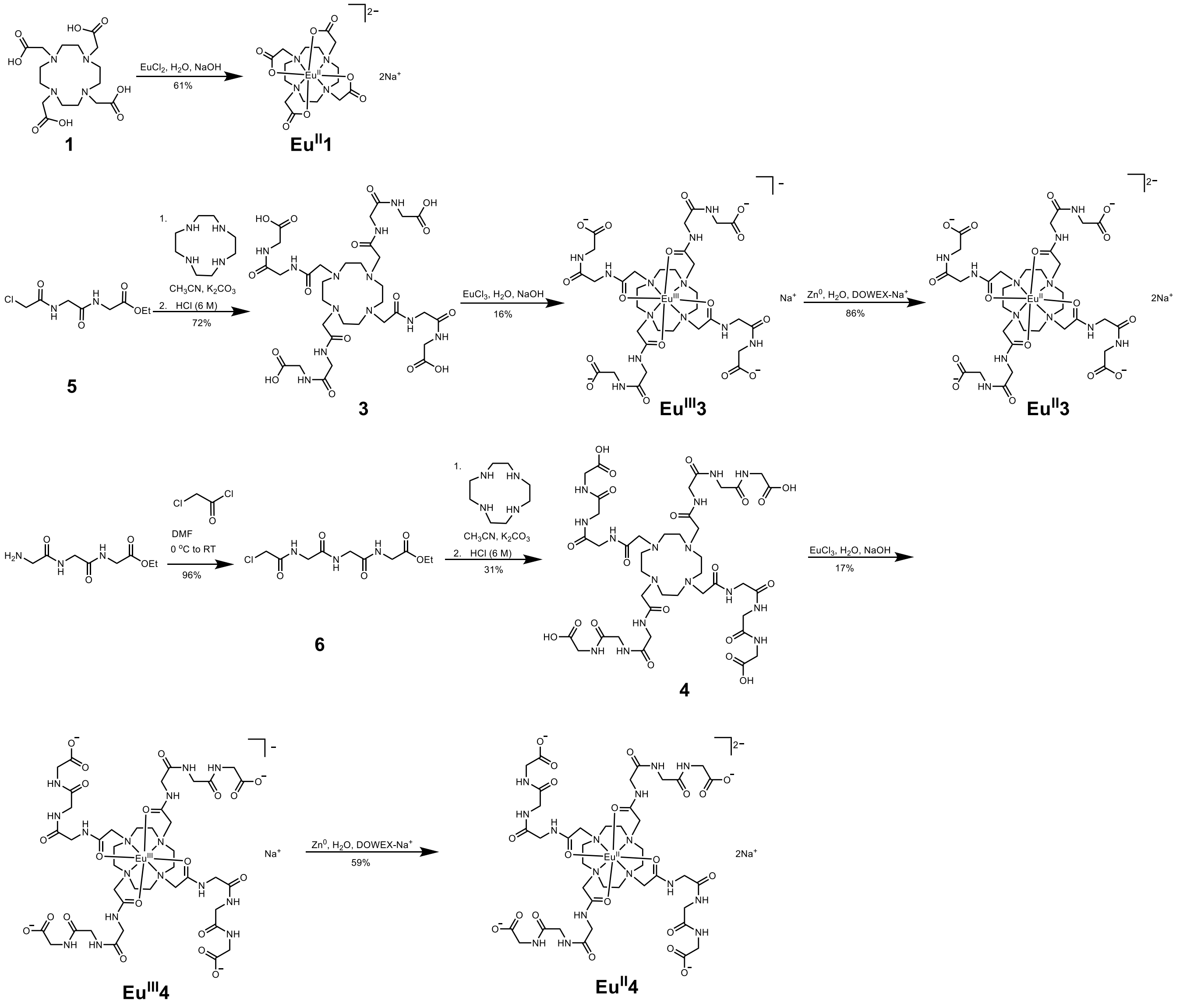

Scheme S1. Synthetic routes for **Eu^{II}1**, **Eu^{II}3**, and **Eu^{II}4**.

Absorption and emission studies

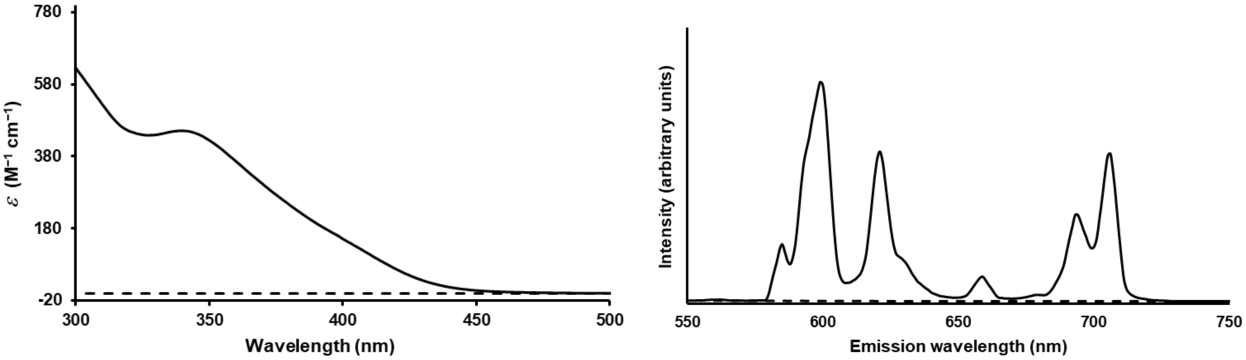

Figure S1. (Left) Molar extinction coefficient spectra (ε) of **Eu^{II}1** (—) and **Eu^{III}1** (**----**) post-oxidation of **Eu^{II}1** by exposure to air. (Right) Emission spectra (excitation at 395 nm) of **Eu^{II}1** (—) and **Eu^{III}1** (**----**) after oxidation of **Eu^{II}1** by exposure to air.

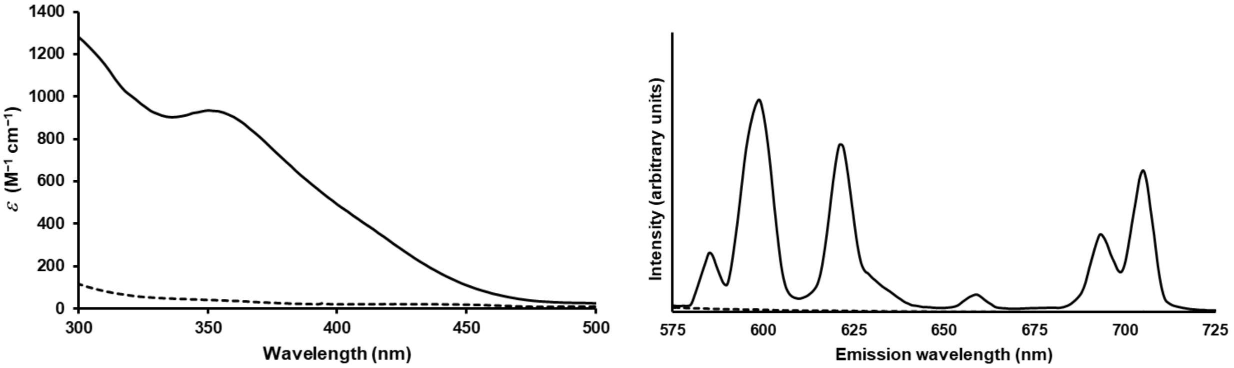

Figure S2. (Left) Molar extinction coefficient spectra (ε) of **Eu^{II}3** (—) and **Eu^{III}3** (**----**) post-oxidation of **Eu^{II}3** by exposure to air. (Right) Emission spectra (excitation at 395 nm) of **Eu^{II}3** (—) and **Eu^{III}3** (**----**) after oxidation of **Eu^{II}3** by exposure to air.

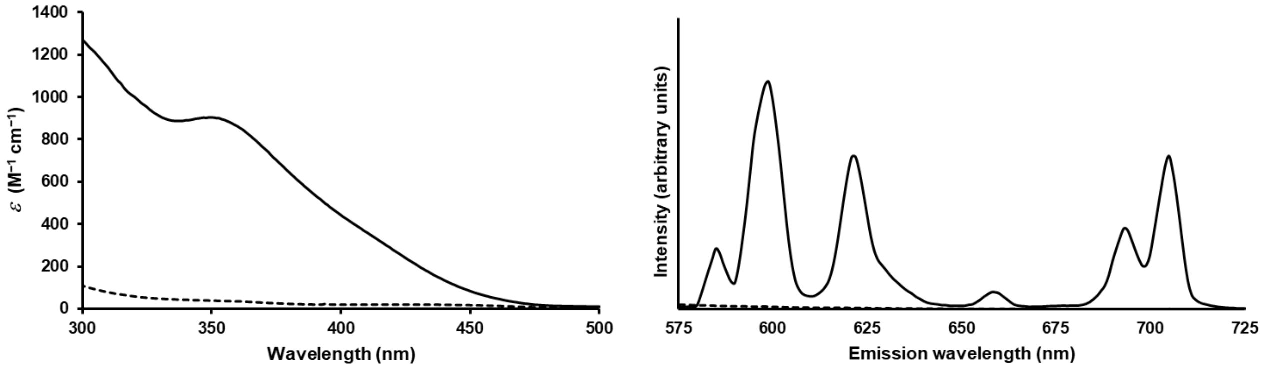

Figure S3. (Left) Molar extinction coefficient spectra (ε) of **Eu^{II}4** (—) and **Eu^{III}4** (**----**) post-oxidation of **Eu^{II}4** by exposure to air. (Right) Emission spectra (excitation at 395 nm) of **Eu^{II}4** (—) and **Eu^{III}4** (**----**) after oxidation of **Eu^{II}4** by exposure to air.

NMR spectra

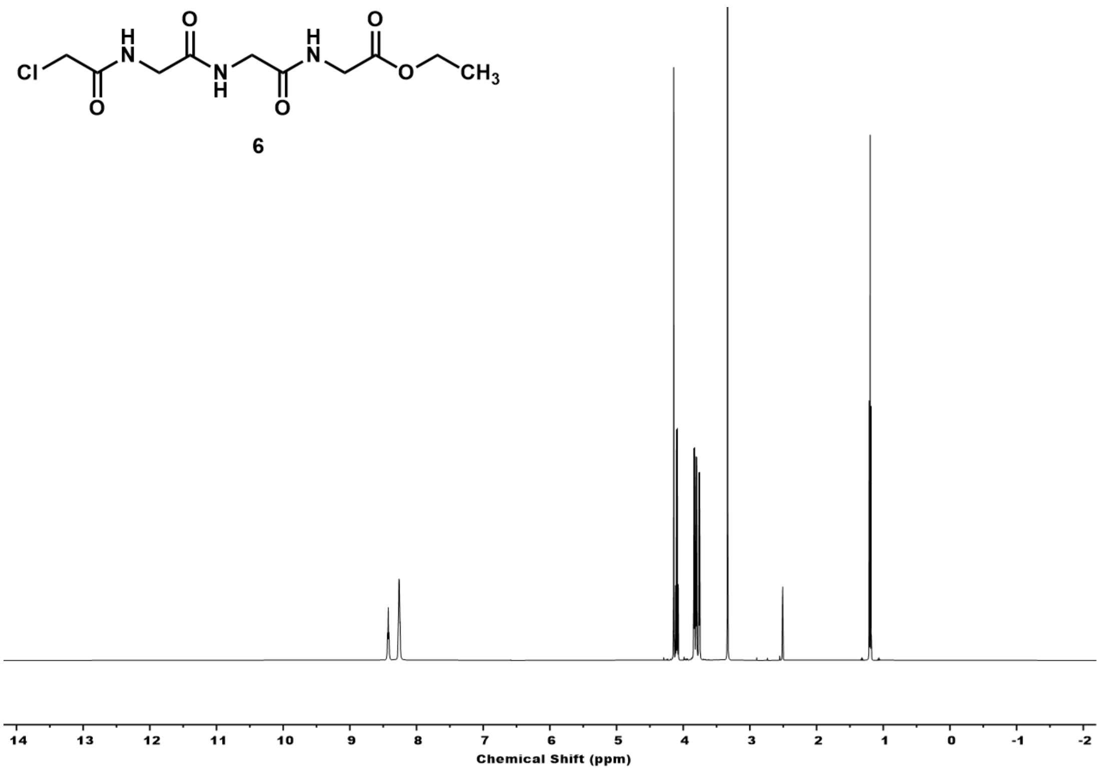

Figure S4. ^{1}H-NMR spectrum of **6**.

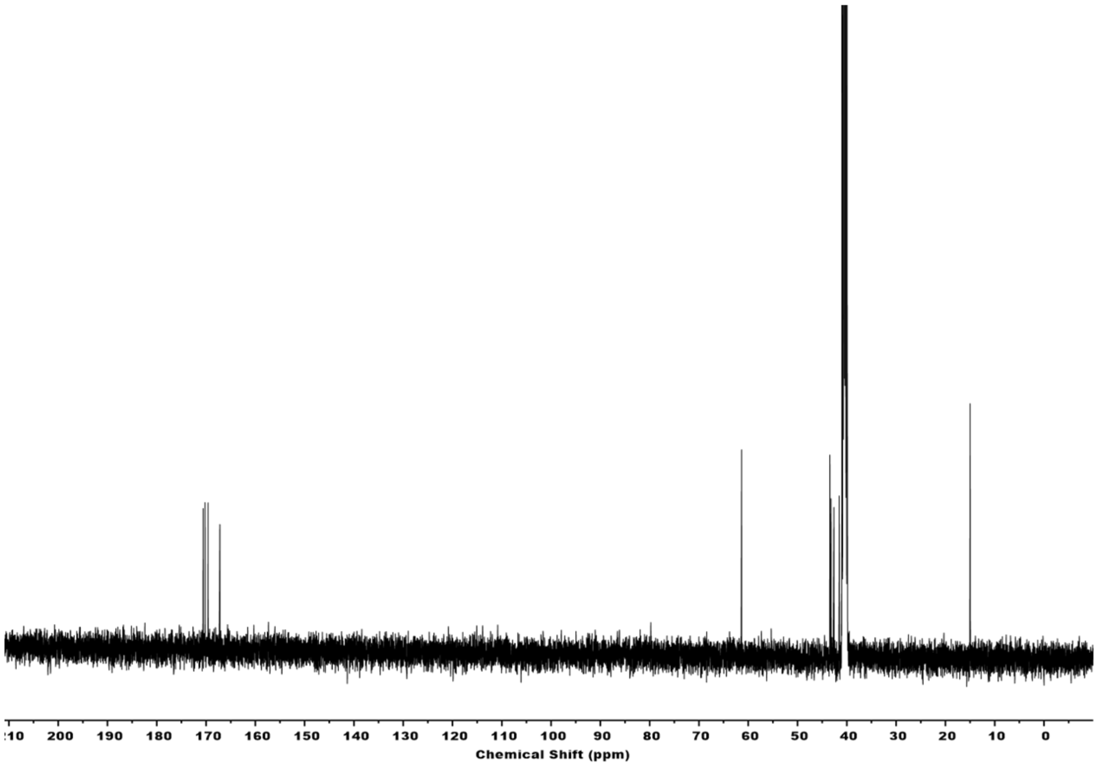

Figure S5. ^{13}C-NMR spectrum of **6**.

Minimum detectable concentration study

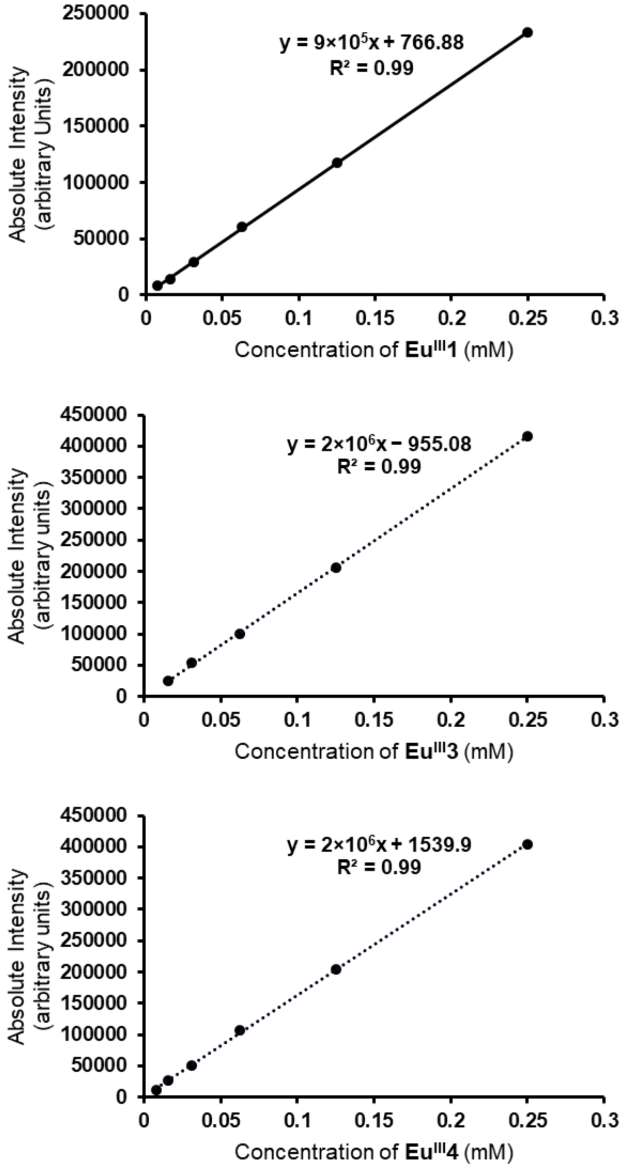

Figure S6. Absolute emission intensity at 587 nm (excitation at 395 nm) versus concentration of **Eu^{III}1**, **Eu^{III}3**, and **Eu^{III}4**. Solid lines represent the best-fit linear trendlines.

Cyclic voltammograms for electrochemical study

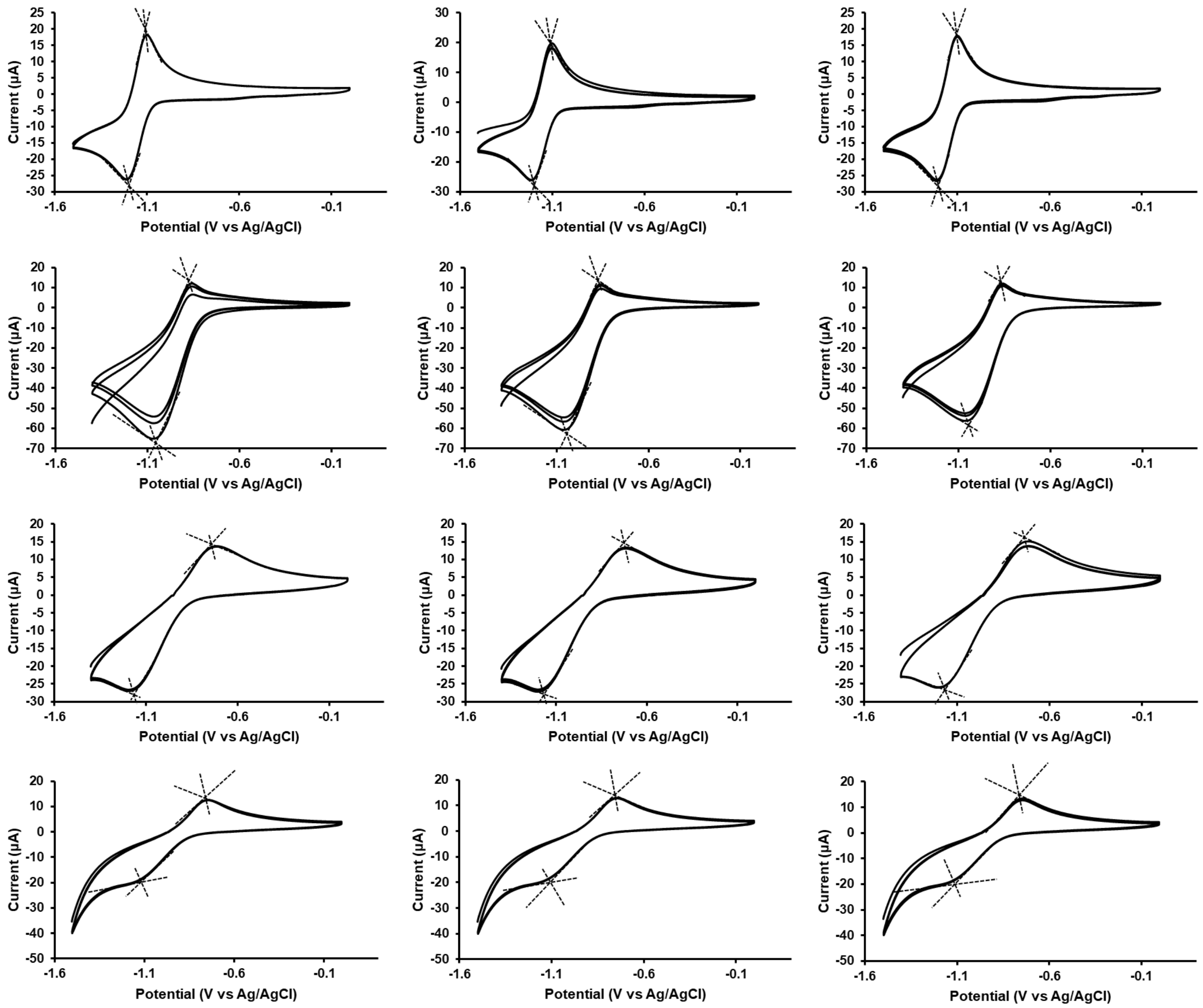

Figure S7. Cyclic voltammograms of **Eu$^{II/III}$1** (1st row), **Eu$^{II/III}$2** (2nd row), **Eu$^{II/III}$3** (3rd row), and **Eu$^{II/III}$4** (4th row). Dotted lines were used to determine the anodic and cathodic peak potentials for calculating $E_{1/2}$. Experiments were reported three times (3 columns) with independently prepared samples.

Table S1. $E_{1/2}$ and maximum possible distance (r) between Eu and negative ligand charges

Redox couples	$E_{1/2}$ (V versus Ag/AgCl)*	Maximum possible distance between Eu ion to negative ligand charge, r (Å)
**$Eu^{II/III}$1**	−1.142±0.005	3.75
**$Eu^{II/III}$2**	−0.964±0.001	7.33
**$Eu^{II/III}$3**	−0.952±0.002	10.8
**$Eu^{II/III}$4**	−0.938±0.003	14.6

*$E_{1/2}$ values are the mean ± standard error for three independently prepared measurements

Cyclic voltammograms for dissociation studies at pH 7

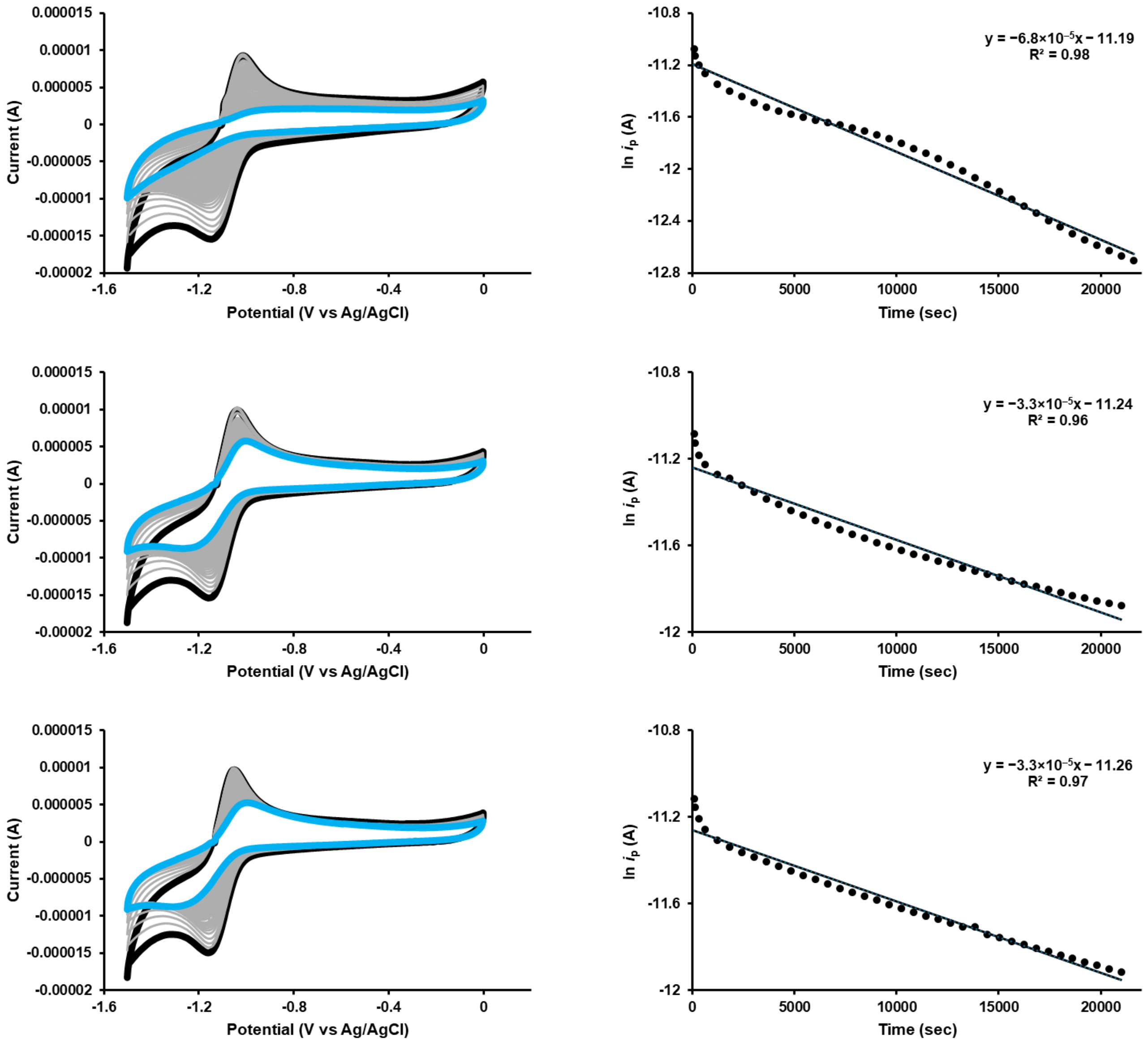

Figure S8. (Left) Cyclic voltammograms of **$Eu^{II/III}$1** . All measurements were performed with solutions (1 mM) of complexes in water at pH 7 with tetraethylammonium perchlorate (100 mM) as the supporting electrolyte. Voltammograms were acquired using 1,402 segments (around 6 h) at 100 mV s^{-1}. Experiments were reported three times (3 rows) with independently prepared samples. The black trace indicates t = 0 h, grey traces are times between t = 0 h and t = 6 h, and the blue trace indicates t = 6 h. (Right) Peak heights of complexed Eu^{II} were used to measure peak current of Eu^{II} at different time points, and the data are plotted against time (dotted lines). Solid lines represent the best-fit linear trendlines.

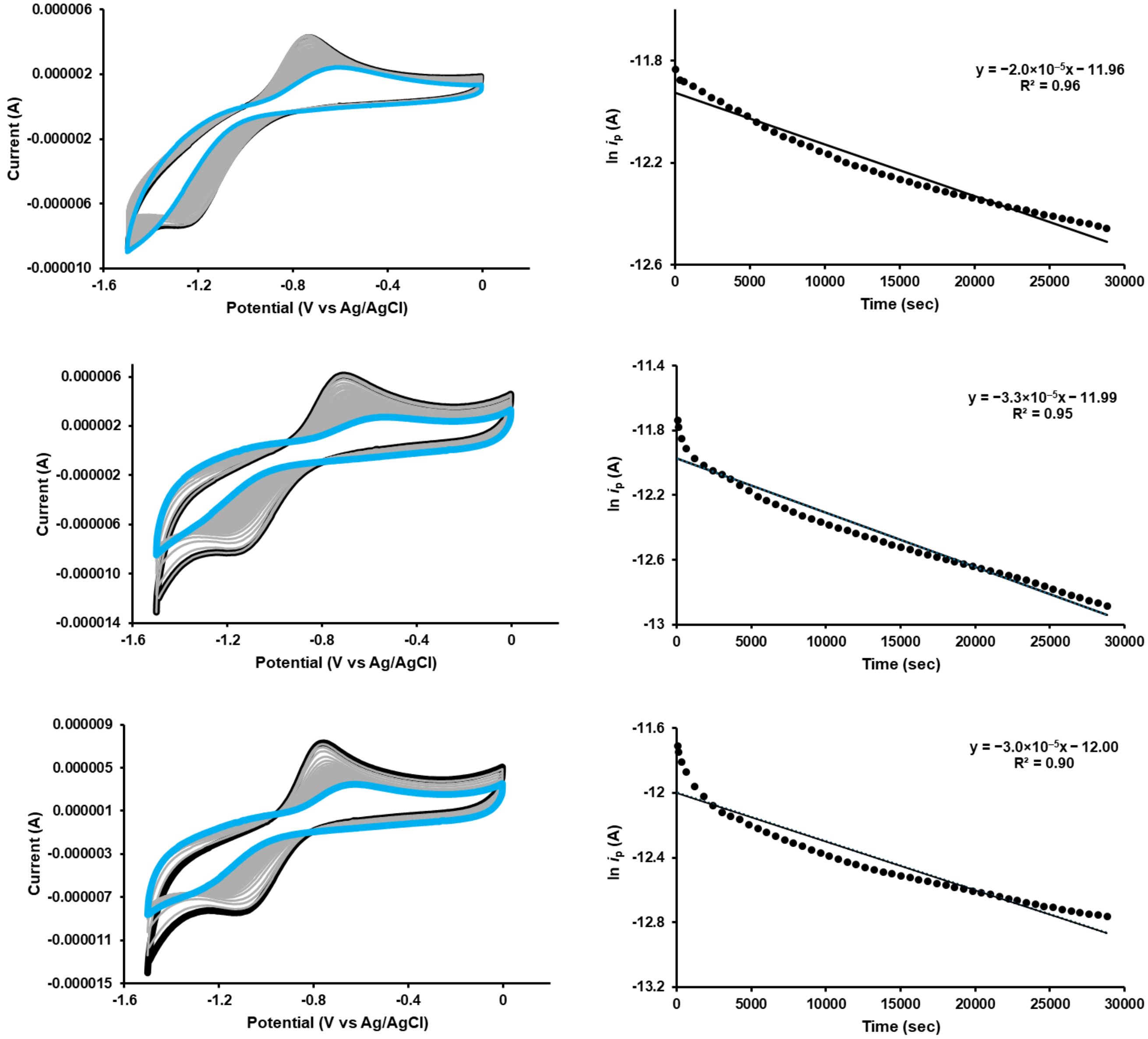

Figure S9. (Left) Cyclic voltammograms of **Eu$^{II/III}$2**. All measurements were performed with solutions (1 mM) of complexes in water at pH 7 with tetraethylammonium perchlorate (100 mM) as the supporting electrolyte. Voltammograms were acquired using 1,950 segments (8 h) at 100 mV s^{-1}. Experiments were reported three times (3 rows) with independently prepared samples. The black trace indicates t = 0 h, grey traces are times between t = 0 h and t = 8 h, and the blue trace indicates t = 8 h. (Right) Peak heights of complexed Eu^{II} were used to measure peak current of Eu^{II} at different time points, and the data are plotted against time (dotted lines). Solid lines represent the best-fit linear trendlines.

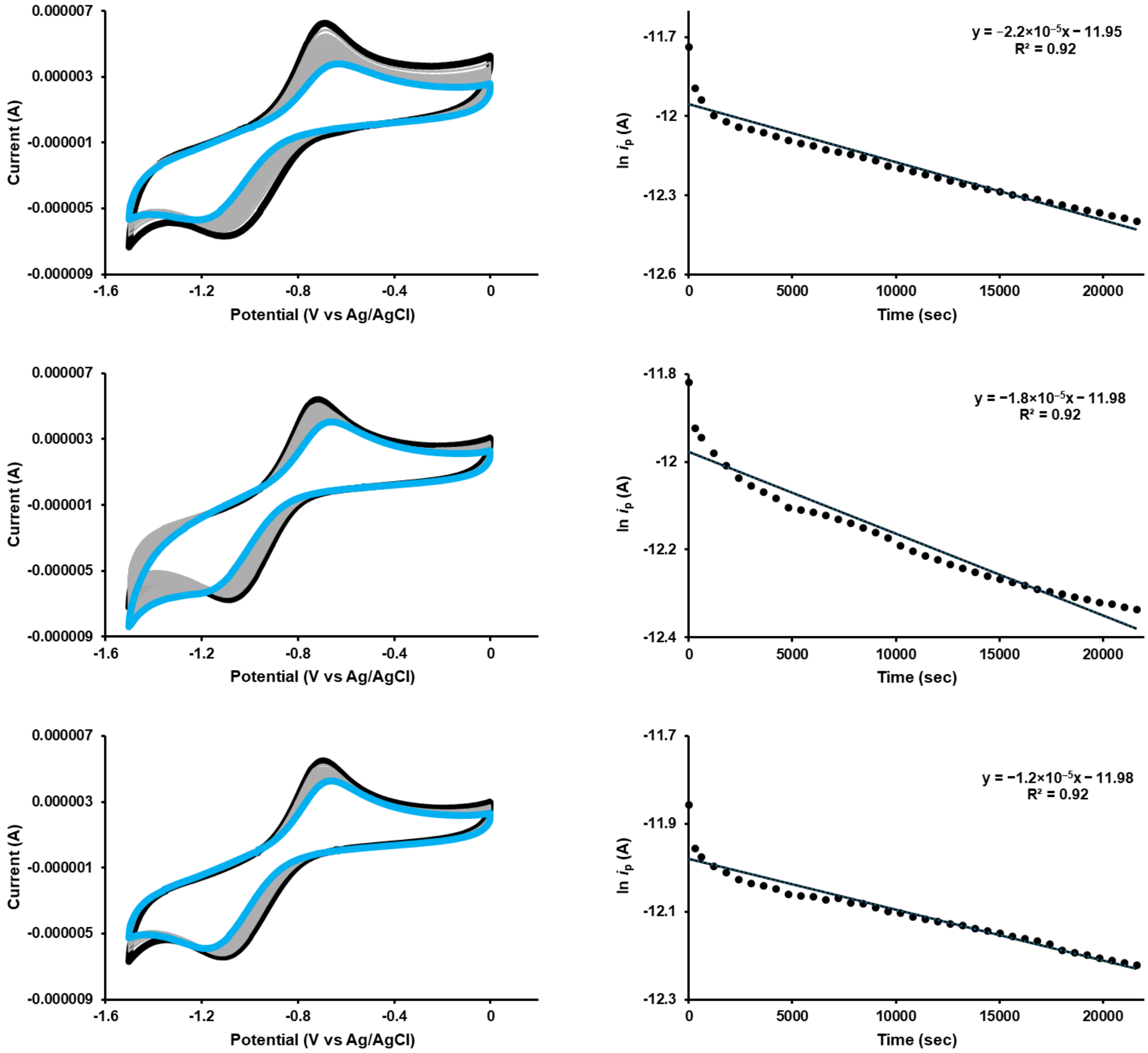

Figure S10. (Left) Cyclic voltammograms of **Eu$^{II/III}$3**. All measurements were performed with solutions (1 mM) of complexes in water at pH 7 with tetraethylammonium perchlorate (100 mM) as the supporting electrolyte. Voltammograms were acquired using 1,450 segments (6 h) at 100 mV s^{-1}. Experiments were reported three times (3 rows) with independently prepared samples. The black trace indicates t = 0 h, grey traces are times between t = 0 h and t = 6 h, and the blue trace indicates t = 6 h. (Right) Peak heights of complexed Eu^{II} were used to measure peak current of Eu^{II} at different time points, and the data are plotted against time (dotted lines). Solid lines represent the best-fit linear trendlines.

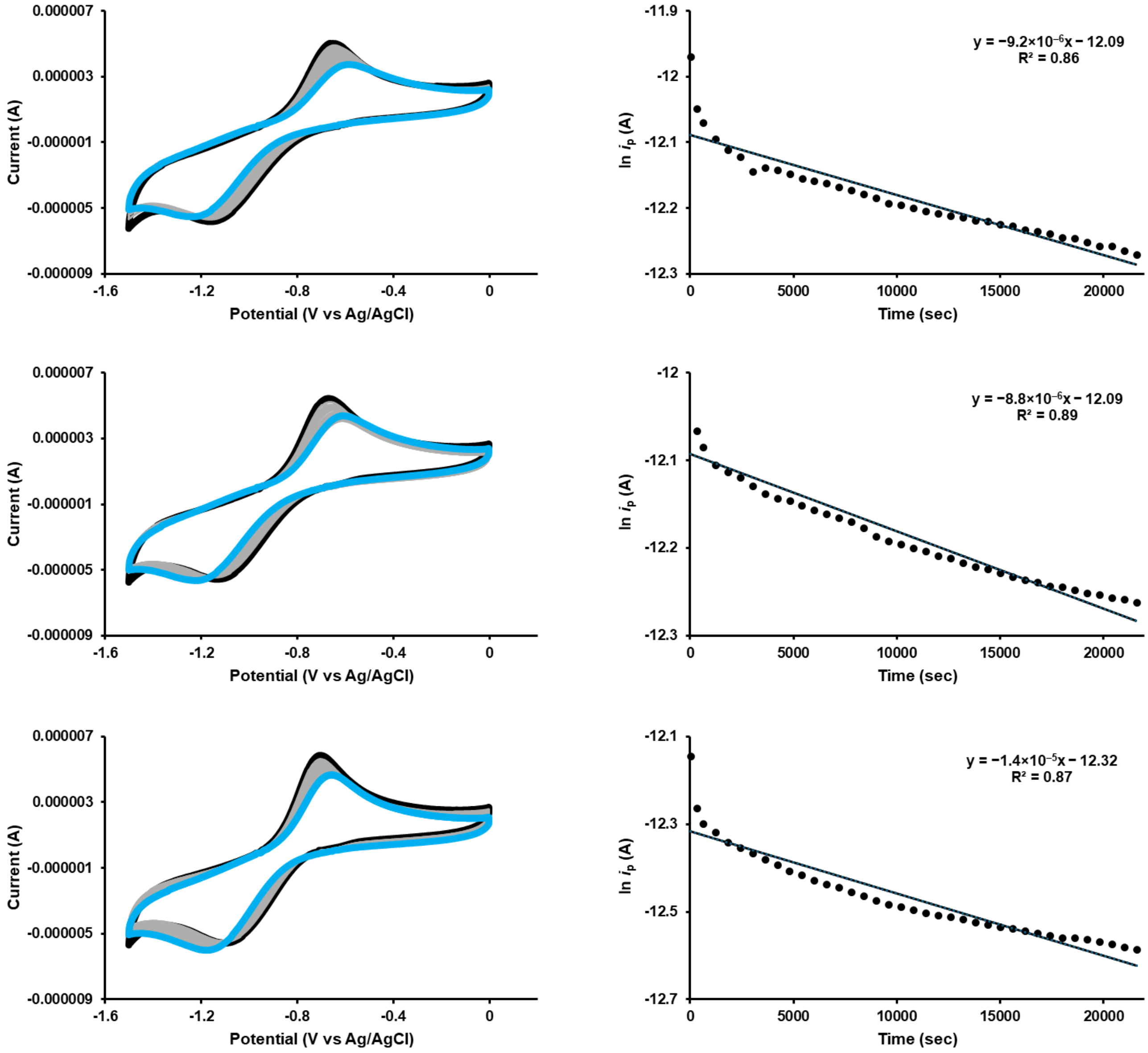

Figure S11. (Left) Cyclic voltammograms of **Eu$^{II/III}$4**. All measurements were performed with solutions (1 mM) of complexes in water at pH 7 with tetraethylammonium perchlorate (100 mM) as the supporting electrolyte. Voltammograms were acquired using 1,450 segments (6 h) at 100 mV s^{-1}. Experiments were reported three times (3 rows) with independently prepared samples. The black trace indicates t = 0 h, grey traces are times between t = 0 h and t = 6 h, and the blue trace indicates t = 6 h. (Right) Peak heights of complexed Eu^{II} were used to measure peak current of Eu^{II} at different time points, and the data are plotted against time (dotted lines). Solid lines represent the best-fit linear trendlines.

Absorption data for dissociation studies at pH 1

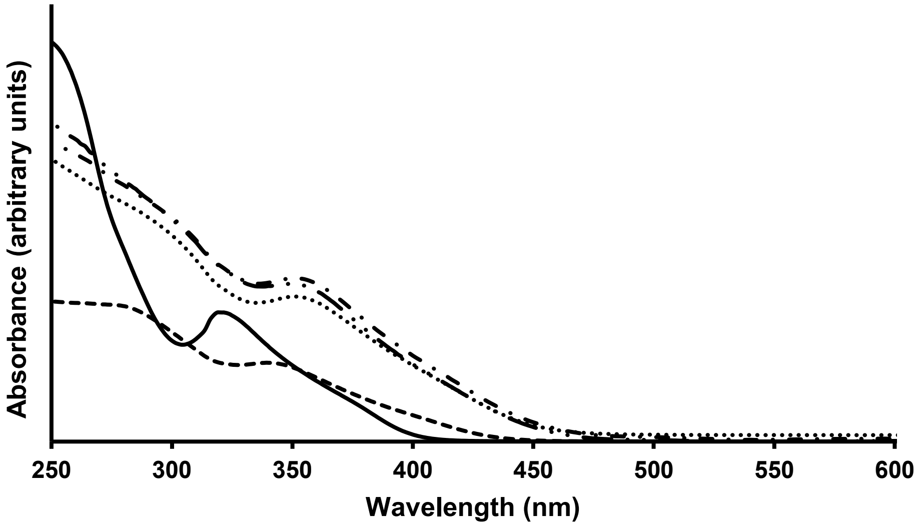

Figure S12. UV−visible absorption spectra of **Eu^{II}1** (----), **Eu^{II}2** (••••••), **Eu^{II}3** (−·−), **Eu^{II}4** (−··−), and $EuCl_2$ (——). All solutions contained the complex (1 mM) in degassed water under an atmosphere of N_2.

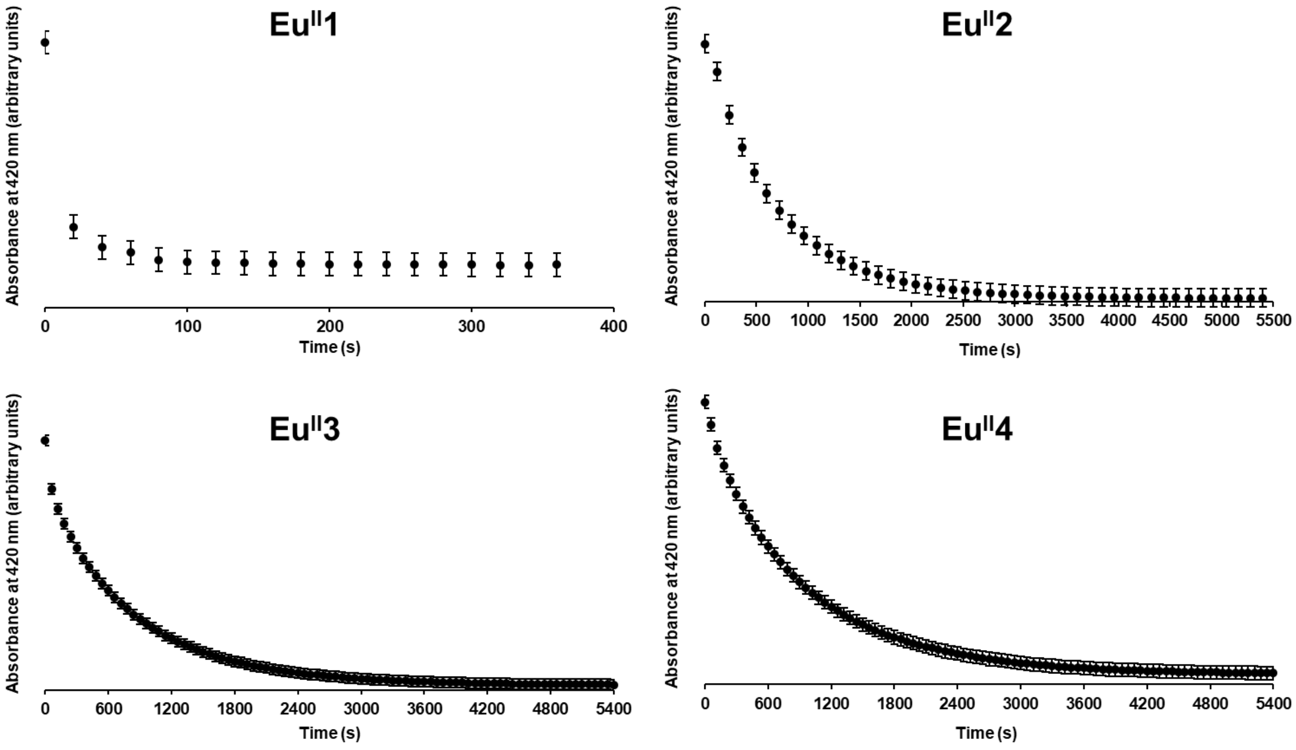

Figure S13. Absorption of **Eu^{II}1**–**Eu^{II}4** at 420 nm as a function of time in aqueous HCl (0.1 M). Error bars represent the standard error of the mean of three independently prepared samples.

Thermogravimetric analyses

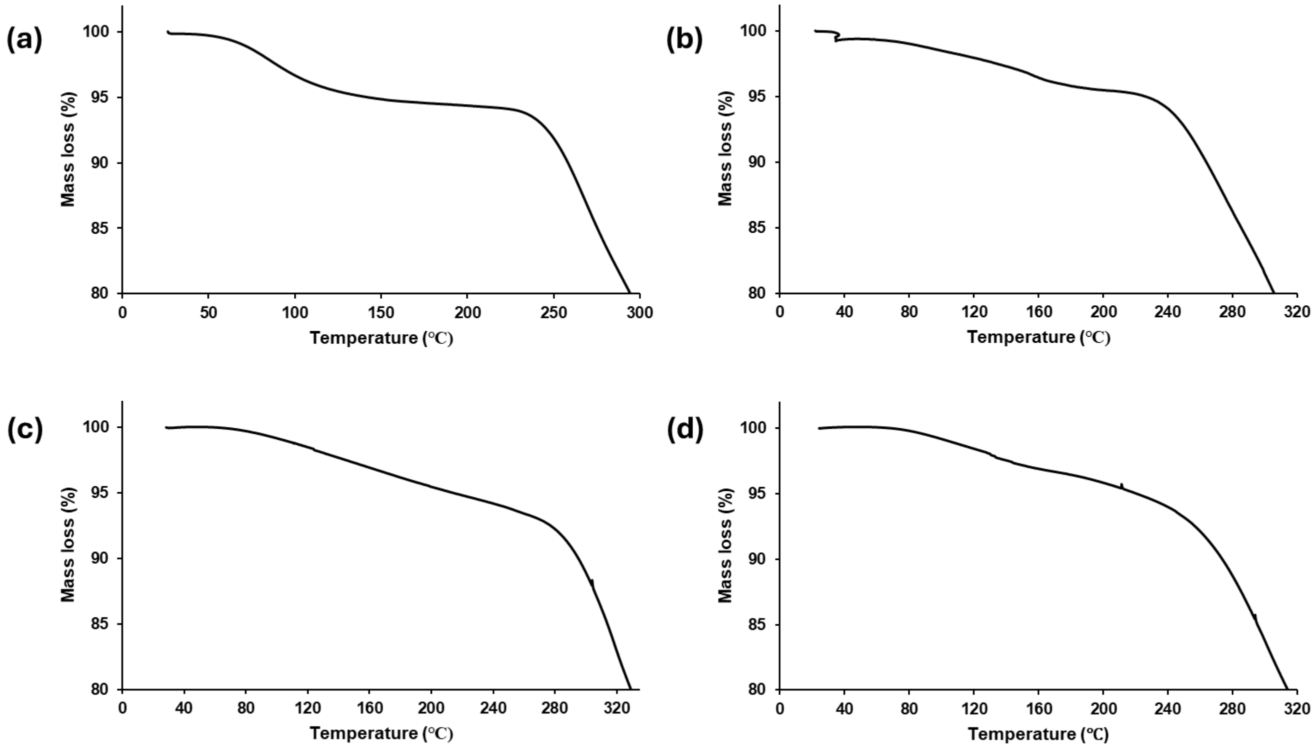

Figure S14. Thermogravimetric analyses of (a) **3**, (b) **4**, (c) $\mathbf{Eu^{III}3}$, and (d) $\mathbf{Eu^{III}4}$ recorded at 10 °C/min under flowing Ar.

Statistical analyses

Table S2. Unpaired *t*-tests of the electrochemical potentials ($E_{1/2}$) of **Eu$^{II/III}$2** versus **Eu$^{II/III}$3** and **Eu$^{II/III}$3** versus **Eu$^{II/III}$4**.

	**Eu$^{II/III}$2**	**Eu$^{II/III}$3**
Mean	0.964	0.952
Variance	6.30×10^{-6}	3.17×10^{-5}
Observations	3	3
P (T ≤ t) two-tail	0.03	
Different ($P < 0.05$)	Yes	
	**Eu$^{II/III}$3**	**Eu$^{II/III}$4**
Mean	0.952	0.938
Variance	3.17×10^{-5}	2.16×10^{-6}
Observations	3	3
P (T ≤ t) two-tail	0.01	
Different ($P < 0.05$)	Yes	

Table S3. Unpaired *t*-tests of the k_d of **Eu^{II}1** versus **Eu^{II}2**, **Eu^{II}1** versus **Eu^{II}3**, **Eu^{II}1** versus **Eu^{II}4**, **Eu^{II}2** versus **Eu^{II}3**, **Eu^{II}2** versus **Eu^{II}4**, and **Eu^{II}3** versus **Eu^{II}4**.

	**Eu^{II}1**	**Eu^{II}2**
Mean	4.50×10^{-5}	2.78×10^{-5}
Variance	4.08×10^{-10}	4.81×10^{-11}
Observations	3	3
P (T ≤ t) two-tail	0.24	
Different ($P < 0.05$)	No	
	**Eu^{II}1**	**Eu^{II}3**
Mean	4.50×10^{-5}	1.72×10^{-5}
Variance	4.08×10^{-10}	2.59×10^{-11}
Observations	3	3
P (T ≤ t) two-tail	0.08	
Different ($P < 0.05$)	No	
	**Eu^{II}1**	**Eu^{II}4**
Mean	4.50×10^{-5}	1.08×10^{-5}
Variance	4.08×10^{-10}	9.51×10^{-12}
Observations	3	3
P (T ≤ t) two-tail	0.04	
Different ($P < 0.05$)	Yes	
	**Eu^{II}2**	**Eu^{II}3**
Mean	4.50×10^{-5}	1.72×10^{-5}
Variance	4.08×10^{-10}	2.59×10^{-11}
Observations	3	3
P (T ≤ t) two-tail	0.10	
Different ($P < 0.05$)	No	
	**Eu^{II}2**	**Eu^{II}4**
Mean	4.50×10^{-5}	1.08×10^{-5}
Variance	4.08×10^{-10}	9.51×10^{-12}
Observations	3	3
P (T ≤ t) two-tail	0.02	
Different ($P < 0.05$)	Yes	
	**Eu^{II}3**	**Eu^{II}4**
Mean	1.72×10^{-5}	1.08×10^{-5}
Variance	2.59×10^{-11}	9.51×10^{-12}
Observations	3	3
P (T ≤ t) two-tail	0.13	
Different ($P < 0.05$)	No	

Table S4. Unpaired *t*-tests of the k_d at pH 1 of **Eu^{II}2** versus **Eu^{II}3**, **Eu^{II}2** versus **Eu^{II}4**, and **Eu^{II}3** versus **Eu^{II}4**.

	**Eu^{II}2**	**Eu^{II}3**
Mean	13.2×10^{-4}	12.5×10^{-4}
Variance	6.70×10^{-9}	4.75×10^{-9}
Observations	3	3
P (T ≤ t) two-tail	0.3	
Different (*P* < 0.05)	No	
	**Eu^{II}2**	**Eu^{II}4**
Mean	13.2×10^{-4}	11.9×10^{-5}
Variance	6.70×10^{-9}	5.87×10^{-10}
Observations	3	3
P (T ≤ t) two-tail	0.06	
Different (*P* < 0.05)	No	
	**Eu^{II}3**	**Eu^{II}4**
Mean	12.5×10^{-4}	11.9×10^{-5}
Variance	4.75×10^{-9}	5.87×10^{-10}
Observations	3	3
P (T ≤ t) two-tail	0.2	
Different (*P* < 0.05)	No	
